# An anchored chromosome‐scale genome assembly of spinach improves annotation and reveals extensive gene rearrangements in euasterids

**DOI:** 10.1002/tpg2.20101

**Published:** 2021-06-10

**Authors:** Amanda M Hulse‐Kemp, Hamed Bostan, Shiyu Chen, Hamid Ashrafi, Kevin Stoffel, Walter Sanseverino, Linzhou Li, Shifeng Cheng, Michael C. Schatz, Tyler Garvin, Lindsey J. du Toit, Elizabeth Tseng, Jason Chin, Massimo Iorizzo, Allen Van Deynze

**Affiliations:** ^1^ Department of Plant Sciences University of California Davis CA USA; ^2^ USDA Agricultural Research Service, Genomics and Bioinformatics Research Unit Raleigh NC USA; ^3^ Department of Crop and Soil Science North Carolina State University Raleigh NC USA; ^4^ Plants for Human Health Institute North Carolina State University Kannapolis NC USA; ^5^ Department of Horticulture North Carolina State University Raleigh NC USA; ^6^ Sequentia Biotech SL Barcelona Catalonia Spain; ^7^ BGI‐Shenzhen Shenzhen China; ^8^ Agricultural Genomics Institute at Shenzhen Chinese Academy of Agricultural Sciences Shenzhen 518060 P. R. China; ^9^ Cold Spring Harbor Laboratory One Bungtown Road, Koch Building 1121 Cold Spring Harbor NY 11724 USA; ^10^ Departments of Computer Science and Biology Johns Hopkins University 3400 N Charles St Baltimore MD 21218 USA; ^11^ Washington State University SU Mount Vernon Northwestern Washington Research & Extension Center (NWREC) Mount Vernon WA 98273 USA; ^12^ Pacific Biosciences Menlo Park CA USA; ^13^ DNAnexus Inc 1975 W El Camino Real #204 Mountain View CA 94040 USA

## Abstract

Spinach (*Spinacia oleracea* L.) is a member of the Caryophyllales family, a basal eudicot asterid that consists of sugar beet (*Beta vulgaris* L. subsp. *vulgaris*), quinoa (*Chenopodium quinoa* Willd.), and amaranth (*Amaranthus hypochondriacus* L.). With the introduction of baby leaf types, spinach has become a staple food in many homes. Production issues focus on yield, nitrogen‐use efficiency and resistance to downy mildew (*Peronospora effusa*). Although genomes are available for the above species, a chromosome‐level assembly exists only for quinoa, allowing for proper annotation and structural analyses to enhance crop improvement. We independently assembled and annotated genomes of the cultivar Viroflay using short‐read strategy (Illumina) and long‐read strategies (Pacific Biosciences) to develop a chromosome‐level, genetically anchored assembly for spinach. Scaffold N50 for the Illumina assembly was 389 kb, whereas that for Pacific BioSciences was 4.43 Mb, representing 911 Mb (93% of the genome) in 221 scaffolds, 80% of which are anchored and oriented on a sequence‐based genetic map, also described within this work. The two assemblies were 99.5% collinear. Independent annotation of the two assemblies with the same comprehensive transcriptome dataset show that the quality of the assembly directly affects the annotation with significantly more genes predicted (26,862 vs. 34,877) in the long‐read assembly. Analysis of resistance genes confirms a bias in resistant gene motifs more typical of monocots. Evolutionary analysis indicates that *Spinacia* is a paleohexaploid with a whole‐genome triplication followed by extensive gene rearrangements identified in this work. Diversity analysis of 75 lines indicate that variation in genes is ample for hypothesis‐driven, genomic‐assisted breeding enabled by this work.

## Core Ideas

- Quality of genome assemblies directly affect quality of annotation.
- Analysis of resistance genes confirms a bias in resistant gene motifs more typical of monocots.
- *Spinacia* is a paleohexaploid with extensive gene rearrangements.
- Variation in genes is ample for hypothesis‐driven genomic‐assisted breeding.

**Abbreviations**
BUSCO: benchmarking universal single‐copy orthologs
MPE: mate‐pair
MYA: million years ago
PCA: principal component analysis
PE: paired end
SNP: single nucleotide polymorphism
SRS: skim resequencing
WGD: whole‐genome duplication

## 1 INTRODUCTION

Spinach (*Spinacia oleracea* L.) is a diploid, annual, dioecious crop bred for three different commodity markets: fresh market clipped and bagged, fresh market bunched, and frozen. Overall, these markets comprise a total value worldwide of US$7.85 billion in 2009 (http://faostat.fao.org/). For crop improvement, selection for traits depends on market type including leaf type (smooth, semi‐savoy, or savoy), leaf shape, bolting resistance, shelf life, disease resistance (Correll et al., 2011), and yield. Resistance to downy mildew (*Peronospora effusa*) is of particular importance because of the ubiquitous nature of this rapidly evolving pathogen in growing regions and the large portion (30–50%) of the market being organic, restricting chemical control. Biologically, the dioecious mating system is modified by genes conferring monoeciocy. Furthermore, spinach is one of the most nutrient‐dense leafy greens, providing a rich source of vitamins and antioxidants (β‐carotene, lutein, Vitamin C, K, and folate) and minerals (calcium, iron, potassium, magnesium, and manganese).

Spinach is part of the basal branch of Euroasterids Amaranthaceae family, Chenopodiaceae, Caryophyllales, with 2*n* = 12 chromosomes and several crossable wild relatives and germplasm collections (van Treuren et al., 2012) used in breeding. Current genomics resources include a chloroplast genome (Schmitz‐Linneweber et al., 2001), an in‐depth transcriptome database with 72,151 unigenes, few genetic maps and genetic markers for quality traits (Ma et al., 2016; Qin et al., 2017; Shi et al., 2017) and disease resistance. A draft genome assembly has been developed but is highly fragmented (contig N50 = 16.0 Kb) and only 47% of the assembled sequences were anchored. Additionally, sequence‐based diversity analysis has been performed on 120 lines providing a single nucleotide polymorphism (SNP) database (Xu et al., 2017). A long‐read, genetically anchored, high‐quality chromosome level genome assembly is essential to fully elucidate and leverage the genes responsible for crop improvement traits and understanding the domestication of this basal asterid. We have developed such a sequence assembly and annotated and genetically anchored it using short‐ and long‐read technologies. We show the benefits of long‐reads to assemble the genome and for annotation of complete gene models. It also serves as key resource to study evolution of this basal Eudicot.

## 2 MATERIALS AND METHODS

### 2.1 Plant material and DNA sequencing of cultivar Viroflay

Ninety‐four plants homozygous for 384 random SNP markers (data not shown) were selected for sequencing from the monoecious spinach heirloom cultivar Viroflay. DNA was extracted using a modified CTAB method (Stoffel et al., 2012). Genomic libraries were created and sequenced for Pacific Biosciences and Illumina for genome sequencing using manufacturer protocols.

Total RNA was extracted from 17 tissues and treatments. RNA sequencing libraries were created for all tissues and sequenced on Illumina HiSeq 2000 and Pacific Biosciences Iso‐Seq libraries were created and sequenced for four libraries on PacBio RSII (Supplemental Table S6).

The Illumina genome reads were assembled using SOAPdenovo version 2.04 (Li et al., 2008) as described in Supplemental Materials and Methods and the Pacific Biosciences reads were assembled using HGAP and Celera assembler software and polished using Quiver (Chin et al., 2013). The gene space between genomes was compared using CoGent (Workman et al., 2018).

To genetically anchor the genome, a population of 77 recombinant inbred lines were sequenced on a Illumina Hi Seq 3000, reads were mapped to the SpoV3.0 assembly and genetically mapped using MSTMAP. Similarly, for diversity analysis, 75 lines were sequenced at 8–10× coverage using Illumina. These were mapped to the PacBio V3.0 assembly and SNPs were called using HaplotypeCaller of GATK version 3.5. Population analysis was conducted in plink v1.9 and R v 3.6.1. to determine principle components and structure using fastStructure version 1.0.

Annotation included repeat analysis and gene model prediction. Repeat analysis was done in a two‐step process including RepeatModeler a de novo repeats identification and annotation pipeline to identify species‐specific repetitive elements followed by RepeatMasker integrating the de novo repeat database and Repbase. Gene models were predicted using a pipeline outlined in Li et al.(2020) that predicts genes using AUGUSTUS v2.5.5 (Stanke et al., 2006) and integrates evidence using MAKER (v.2.31.8) (Cantarel et al., 2008). Putative gene functions were assigned according to the best match of the alignments using Blast (E‐value ≤ 10 × 10^−5^) to SwissProt and TrEMBL databases. The motifs and domains of genes were determined by InterProScan version 4.7 (Zdobnov & Apweiler, 2001). A detailed transcription factor analysis was carried out using PlantTFcat (Dai et al., 2013), and a comprehensive resistance gene analysis was carried out using PRGdb 3.0 (Osuna‐Cruz et al., 2018).

Analyses of collinearity and synteny between SpoV3, Arabidopsis v11 (https://www.arabidopsis.org/download/index‐auto.jsp?dir=/download_files/Genes), grapevine (Jaillon et al., 2007), sugar beet (Funk et al., 2018), and quinoa (Jarvis et al., 2017) was carried out with MCScanX (Wang et al., 2012). A species tree was built in OrthoFinder using whole‐genome protein sets from all five eudicot genomes plus rice (*Oryza sativa* L.) and sorghum [*Sorghum bicolor* (L.) Moench] (Cooper et al., 2019; Kawahara et al., 2013).

**Core Ideas**

- Quality of genome assemblies directly affect quality of annotation.
- Analysis of resistance genes confirms a bias in resistant gene motifs more typical of monocots.
- *Spinacia* is a paleohexaploid with extensive gene rearrangements.
- Variation in genes is ample for hypothesis‐driven genomic‐assisted breeding.

## 3 RESULTS

### 3.1 Sequencing and assembly of the spinach Viroflay genome

Viroflay was selected as the representative line for reference genome sequencing as a monoecious spinach heirloom cultivar with a large smooth leaf type. A total of eight Illumina libraries (three short‐insert paired end [PE], and five mate‐pair, MPE) were generated and sequenced, producing an overall 172.2 Gb of raw data, which was filtered for quality to retain 126.2 Gb (131.9× coverage, Supplemental Table S1). A portion (27.6 Gb) of the PE‐filtered reads were used to estimate genome size using the k‐mer method with the Jellyfish software (Supplemental Figure S1). The k_num value (the count of k‐mers) was found to be 22,948,604,215 and the peak depth is 24 (Supplemental Table S2). The Viroflay genome size was estimated to be 956.2 Mb, which is consistent with earlier flow‐cytometry analysis, 989 Mb (Arumuganathan & Earle, 1991). The filtered PE data was assembled with SOAPdenovo (Li et al., 2008) to produce contigs that were scaffolded with the MPE data to produce the Spov2 Illumina genome assembly. After filling the gaps, the Spov2 resulted in 1,075,770 scaffolds covering 968.8 Mb with an N50 (50% of the genome is in fragments of this length or longer) of 389 kb (Supplemental Table S3) and contig N50 of 21.4 kb.

**FIGURE 1:**
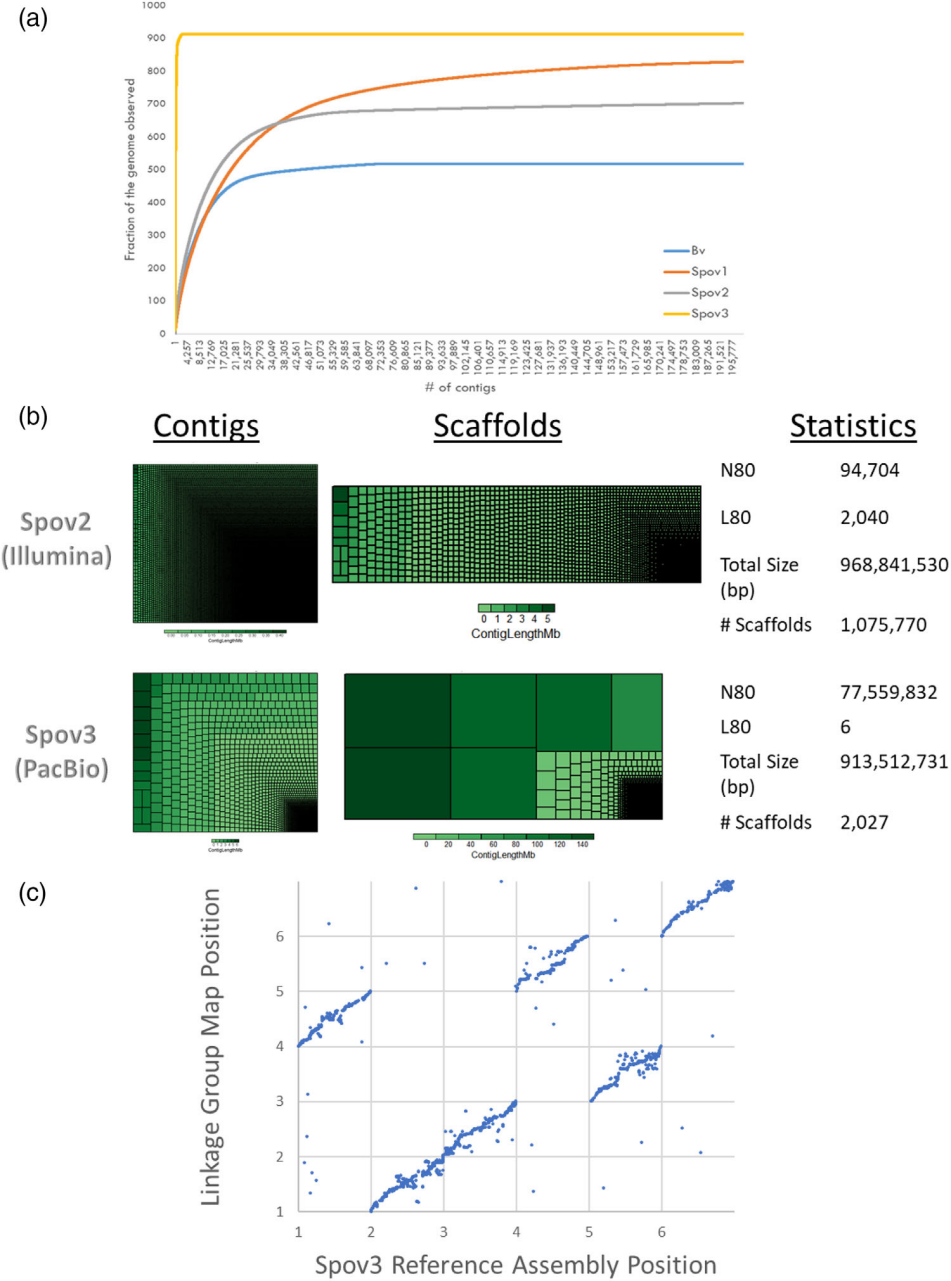
Assessment of contiguity of *Spinacia oleracea* Spov3 assembly compared with others. (a) Portion of assembly based on contig size plot includes *Spinacia oleracea* assembly versions Spov2 (Illumina genome produced as part of this project) and Spov1 (Xu et al., 2017), and sugar beet (*Beta vulgaris* L.) (Dohm et al., 2014), (b) TreeMap of contig and scaffold sizes of Spov2 vs. Spov3. (c) Correlation of resequencing‐based spinach genetic linkage map with the Spinach Reference Assembly (Spov3)

Four Pacific Biosciences libraries were generated and sequenced to a total of ∼70× genome equivalents with 128 SMRT cells. This was assembled de novo with the Celera assembler (Myers et al., 2000), base sequences were polished using the Illumina PE data, and then scaffolded using the Illumina MPE data. A final assembly, Spov3, was produced that resulted in 2,027 scaffolds covering 913.5 Mb with an N50 of 121.9 Mb and contig N50 of 1.8 Mb. This contig N50 represents nearly 110× improvement on both short‐read assemblies and includes ∼83 Mb of additional sequence over the Spov1 (Xu et al., 2017) assembly. The total assembly size represents 92.3–95.5% of the estimated genome size of spinach, depending if using the estimate from k‐mer analysis or prior flow cytometry (Arumuganathan & Earle, 1991). The contiguity of the Spov3 assembly was compared with the sugar beet assembly (Dohm et al., 2014), the available genome sequence for spinach, Spov1, and the Spov2 Illumina assembly produced in this study (Figure 1a; Supplemental Table S3). The Spov3 assembly clearly is much more contiguous, with the six main scaffolds of spinach representing over 80% of the total assembly (L80). The contig and scaffold sizes of the Spov2 and Spov3 assemblies were also compared using TreeMap (Figure 1b).

### 3.2 Genetic linkage map and anchoring the genome

A high‐density genetic map was produced from a population of 77 recombinant inbred lines (F6 RIL) by whole‐genome skim resequencing (SRS) obtaining an average coverage per individual of 3×. The SRS linkage map included six linkage groups, equal to the number of spinach chromosome pairs, with 1,612 SNP bin markers spanning 3,991 cM and associated with 745 Mb, or 81.6%, of the total sequenced genome length with 462 kb average distance between markers (Table 1; Supplemental Tables S4 and S5). Correlation of the final SRS linkage map with the Spov3 pseudomolecules showed high level of collinearity between the linkage map and the genome sequence (Figure 1c). The linkage map anchored 86.7% of the genome, of which, 80% of scaffolds were oriented. The pseudomolecules were assigned chromosome numbers based on length of scaffolds in descending order, except for chromosome 1, which was named based on localization of a sex‐linked simple sequence repeat marker, SO4*. It was uniquely mapped and has been previously associated with the chromosome by in situ hybridization in spinach (Khattak et al., 2006). All following genomic analyses will be evaluating the Spov3 final pseudomolecule assembly unless indicated otherwise.

**TABLE 1:** Summary of high‐density spinach linkage map. Genetic map produced from 77 individuals via single nucleotide polymorphisms identified by whole‐genome resequencing data

Chromosome	Linkage group	Markers	Bins	Distance	Avg. markers per bin	Scaffold no.	v3_chr length	Percentage of genome	Avg. distance per marker
				cM			bp	%	bp
Spov3_chr1	LG04	237	229	631.06	1.03	8	126,466,280	17.38	533,613
Spov3_chr2	LG01	424	342	661.77	1.24	7	153,851,519	19.66	362,857
Spov3_chr3	LG02	319	302	887.23	1.06	9	149,809,010	17.37	469,621
Spov3_chr4	LG05	247	222	1001.01	1.11	7	121,902,013	16.71	493,530
Spov3_chr5	LG03	221	179	368.33	1.23	3	115,496,888	12.59	522,610
Spov3_chr6	LG06	164	157	441.65	1.04	5	77,559,832	9.79	472,926
Pseudomolecules only		1,612	1,431	3,991.06	1.13	6	745,085,542^a^	81.56	462,212
Total		–	–	–	–	26	792,232,018^b^	86.72	475,860

^a^ Lengths include only scaffolds that were placed into the six pseudomolecules.

^b^ Includes each unique scaffold present in the overall map and their total length; Scaffold no. for each linkage group does include redundancy.

### 3.3 Genome assembly quality and analysis of gene families

After confirming the scaffolding accuracy, multiple analyses were performed to verify the quality of the assembly at the base and gene‐scale levels. Average mapping of the PE Illumina transcriptome reads (99.55%) from 17 tissue/stages and PacBio IsoSEquation (99.98%) high‐quality, full‐length transcripts from four tissue samples to the Spov3 genome assembly demonstrated a comprehensive gene space coverage (Supplemental Table S6). No significant sequence contamination was detected. Benchmarking universal single‐copy orthologs (BUSCO) v4 analysis identified 2,017 out of 2,121 (95.1%) complete genes from the core Eudicot gene set, the majority of which (1,955) were single copy (Supplemental Table S7).

Further analysis of the gene space with the PacBio Iso‐Seq data was conducted using COGENT analysis of gene family reconstruction (Tseng, 2018), which produced a total of 8,425 gene families from the data. Mapping of the reconstructed gene family contigs back to the genomes showed the PacBio (99.36%) and Illumina assemblies (99.56%) to have comparable accuracies. It showed that overall, the PacBio genome assembly contained more (7,272) of these gene families present as complete copies than the Illumina (7,063) genome assembly (Supplemental Table S8). This analysis was able to identify 24 gene families that were present on 13 scaffolds in the Illumina assembly (3.593 Mb) but were missing from the PacBio assembly. The longest Illumina scaffold, scaffold17, size 1,353,104, contained 10 of the missing gene families. The contigs from the Illumina genome associated with these genes were extracted and added to the final genome assembly to enhance the gene space (Supplemental Table S9).

### 3.4 Genome annotation and the bias of sequencing technology on gene prediction

The assembled spinach genome Spov3 contains a total of ∼634 Mb (69.44%) of repeat sequences that is slightly higher (+16 Mb) than what was previously estimated in the Spov1 genome sequence (Xu et al., 2017). Class I transposons represent the largest portion of repeat sequences, covering 475 Mb (51.5%) of the genome. Long‐terminal repeat retrotransposons ,are the predominant subgroup in the class I transposon family, occupying 456 Mb (49.9%) of the genome (Supplemental Table S10).

**FIGURE 2:**
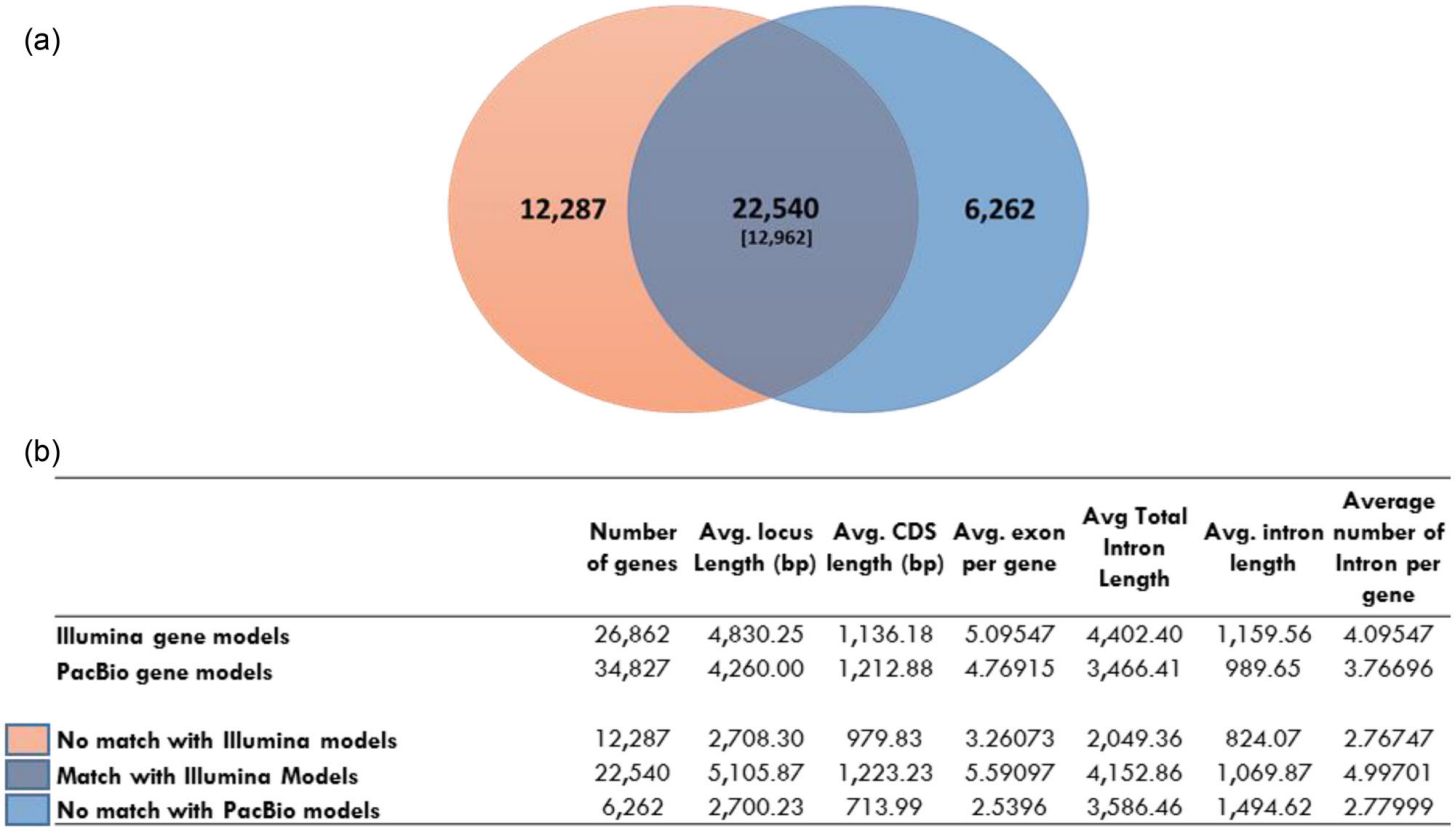
Effect of genome assembly on annotation. (a) The overlapping status of PacBio‐based gene models compared with the Illumina‐based gene models predicted on the Illumina genome. Orange, the gene models unique to the PacBio genome assembly; light blue, the PacBio predicted gene models with a match on the Illumina genome assembly (the subset of genes in parentheses have an exact match in this category); dark blue, the Illumina gene models with no PacBio match. (b) The gene structure statistics for each category. *Note*. 50 genes from the PacBio genome did not map to Illumina sequence and were not included in the analysis

The final annotation yielded 34,877 genes for Spov3. The average coding sequence size was 1,207 bp (Supplemental Table S11), like other annotated genomes, with an average of 4.9 exons per gene. Approximately 92.5% of the genes have either known homologs or can be functionally classified (Supplemental Table S12, Supplemental Figure S2). To further evaluate the quality of the genome sequence assemblies (Spov2 and Spov3), a comprehensive comparative analysis of base‐gene models was carried out (See Materials and Methods section). The Spov2 assembly yielded 26,862 genes, of which 22,694 were functionally annotated. The analysis revealed that 12,287 and 6,262 gene models were unique to PacBio (Spov3) and Ilumina (Spov2), respectively (Figure 2a). Interestingly, sequence of all but 50 PacBio gene models was found in the Spov2 assembly (Figure 2b). Also, 22,540 genes from the PacBio set matched in the Illumina gene models including 12,962 exact matches. To verify the accuracy of the number of gene models, we scanned them for presence of transposons. The scan identified 30 genes in Spov2 and 1,218 genes in Spov3 were transposons, confirming that Spov2 has 26,832 protein coding genes and Spov3 has 33,660 (Supplemental Tables S13 and S14). Although, FAR1 transcription factors contained transposase motifs as expected (Hudson et al., 2003), no transcription factors or resistance genes were identified as transposons.

### 3.5 Transcription factors and resistance genes

PlantTFcat (Dai et al., 2013), a reference plant transcription factor and transcriptional regulator categorization tool, was used to predict the transcription factors and regulatory genes in Spov3 gene models. The analysis identified 3,702 transcription factors (containing 3,887 unique domains) from 20 family types and 98 families in Spov3 gene collections. The result of the analysis and the coordinates of those models on the corresponding genomes are provided in Supplemental Tables S15 through S17. The TF families with greater number of genes predicted in the Spov3 compared with the Spov2 genome included the *CHROMO‐DOMAIN* (+109), *FAR* (+241), and *CCHC(Zn)* (+831) that are involved in multiple critical functions including regulation of the phytochrome A‐mediated light signaling, DNA recognition, RNA packaging, activation of transcription, regulation of apoptosis, and lipid binding.

PRGdb 3.0 (Osuna‐Cruz et al., 2018), a comprehensive platform for prediction and analysis of plant disease resistance genes, was used to predict them in the Spov3. The analysis identified 1,004 candidate disease‐resistant genes (with 2,141 domains) belonging to 15 classes in Spov3 gene models (Supplemental Tables S18 & S19). As previously observed, we confirmed that spinach has a very low number (4) of TNL (toll‐interleukin receptor‐like domain, a nucleotide binding site, and a leucine‐rich repeat) resistant genes, similar to sugar beet (1) (Dohm et al., 2014) and to monocots where this family of resistance genes has been completely lost. This suggests that the TNL family may have largely been lost in a specific lineage like Caryophyllales and Ericales while it expanded in other core eudicots species such as tomato (*Solanum lycopersicum* L.), potato (*Solanum tuberosum* L.), grapevine, and Arabidopsis (Iorizzo et al., 2016). Interestingly, TNLs have been associated with broad‐spectrum resistance to pathogens (Claverie et al., 2011; Menz et al., 2018).

### 3.6 Population analysis—resequencing

A set of 75 diverse spinach lines, including cultivars and lines from the USDA germplasm collection, representing all spinach market classes and leaf types (19 smooth, 22 oriental, 9 savoy, 23 semi‐savoy, 2 untested) based on phenotyping in Davis, CA, (Supplemental Table S20) were resequenced with Illumina to generate approximately 8–10× genome equivalents of PE data. This produced a total of 553,615 high‐quality SNPs with <20% missing data per SNP.

**FIGURE 3:**
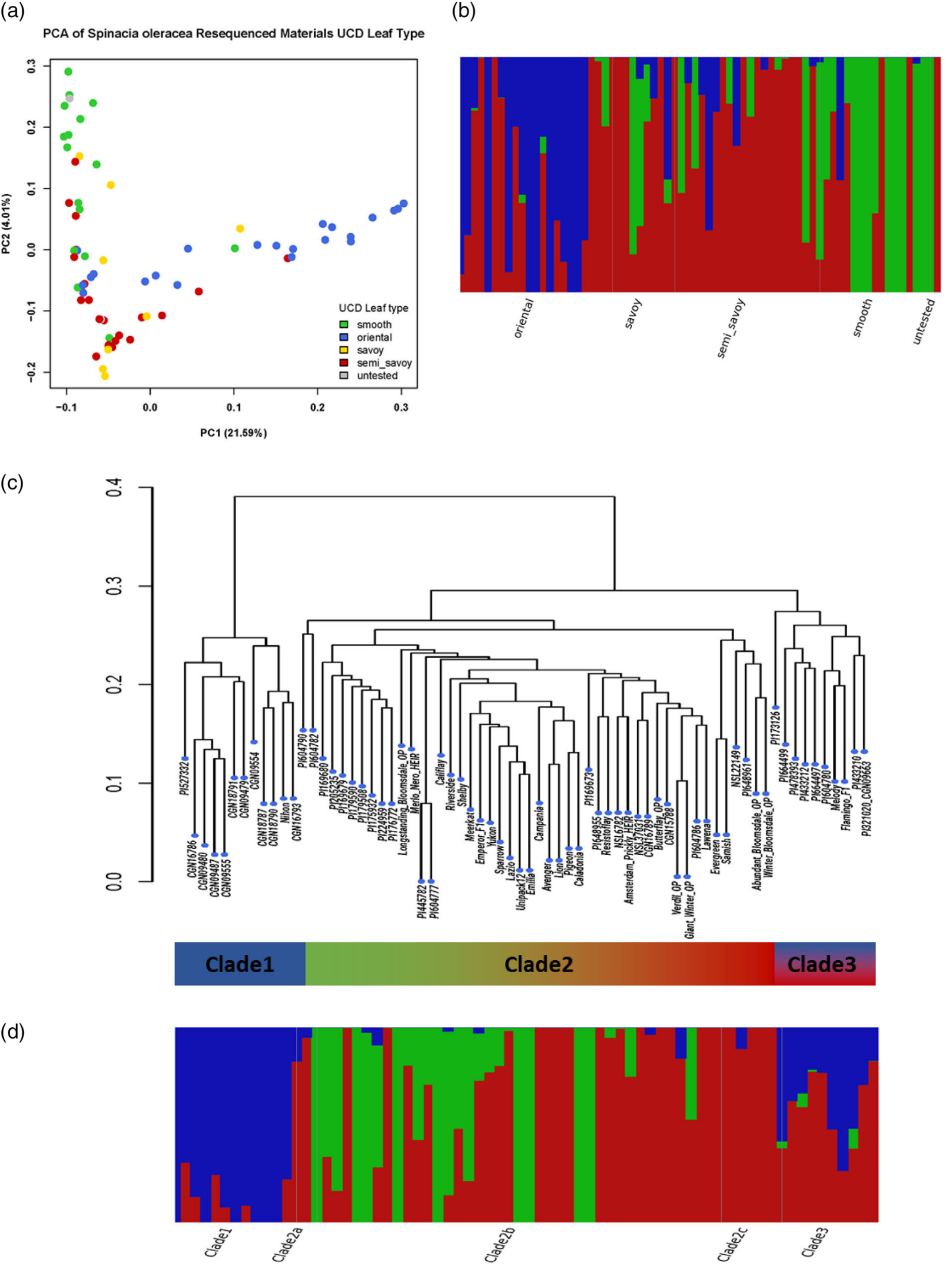
Population analysis of 70 resequenced spinach lines. Labels are indicated for leaf type based on phenotyping in Davis, CA, of materials used for resequencing. (a) Principal component analysis of the first two components PC1 and PC2. (b) Structure analysis with *K* = 3. The *y* axis quantifies cluster membership; the *x* axis represents the different lines. Groups are indicated based on leaf type. (c) Phylogenetic tree of the population based on identity by state, generated with 536,077 high‐quality single nucleotide polymorphisms. (d) Structure analysis with *K* = 3 with groups indicated based on clade in phylogenetic tree shown in (c)

The set of SNPs was used to further study the population structure of spinach after removing five individuals that had >20% missing data (PI374233, ‘Seaside’, ‘Carmel’, ‘Whale’, ‘Clermont’). We performed principal component analysis (PCA, Figure 3a), population‐structure analysis (Figure 3b), and phylogenetic‐tree analysis (Figure 3c). The accessions were classified into four groups corresponding to the four main leaf types (Supplemental Table S20) and used to label the samples in Figure 3. While there were four main leaf types, the population‐structure analysis identified three significant clusters. The phylogenetic analysis also appeared to identify three main clades and these clade definitions were used to label and visualize the population‐structure analysis (Figure 3D).

The PCA produced a triangle‐type distribution of samples but they were not clearly separated into three distinct clusters. The primary principal component was by far the largest, explaining 21.59% of the sample variance, and appeared to separate the oriental type toward the right. Principal component 2 explained 4.01% of the variance but appeared to separate smooth from the savoy and semi‐savoy types. These results were compared with a recent study by Hayes et al. (2020) which also looked at leaf phenotype components for 65 of the 75 lines used in this study (Supplemental Figure S3). The smooth phenotype overlapped with the oriental type in our study.

### 3.7 Ancient whole‐genome triplication and chromosome reconstruction

**FIGURE 4:**
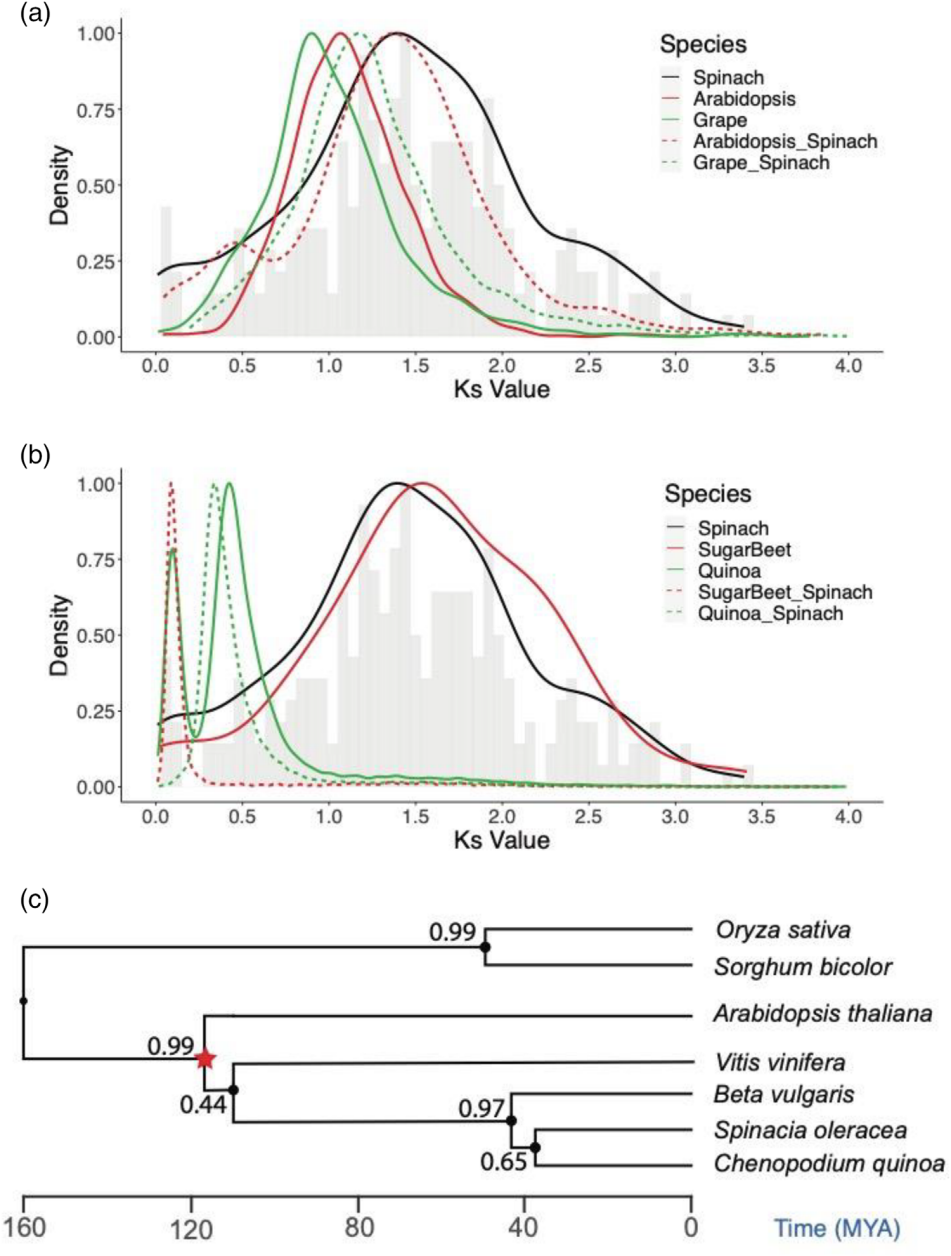
Dating the ancient whole‐genome duplication event in spinach. (a) Distribution of *Ks* values between anchored genes within the collinear blocks within and between genomes of spinach, Arabidopsis, and grapevine. The gray histogram shows the *Ks* value distribution within the spinach genome. The solid and dotted lines represent the kernel density estimation of the *Ks* distributions for intra‐ and intergenomes respectively. (b) *Ks* distribution of spinach, sugar beet, and quinoa. (c) The age estimation of the whole‐genome duplication in spinach. The estimated time of duplication was marked by the red star on the species tree

Inter‐ and intragenome collinear blocks were identified in five eudicot genomes: spinach, grapevine, Arabidopsis, sugar beet, and quinoa. The number of synonymous substitutions per site (*Ks*) in spinach were calculated for 291 anchor gene pairs, and the distribution of *Ks* peaked at value of 1.5, coinciding with the peak of *Ks* distribution calculated between spinach and Arabidopsis (Figure 4a). This suggests that the whole‐genome duplications (WGDs) found in spinach is as old as the divergence between the lineage of Arabidopsis (Rosid) and the lineage of spinach (Euasterid) and is much older than the divergence among the *Armaranthaceae* species, since the *Ks* values between sugar beet, quinoa, and spinach centered around 0.1 to 0.5 (Figure 4b). Based on the mean rate of synonymous substitution (λ) estimated, 6.1 × 10^−9^ in Arabidopsis, the collinear blocks in spinach dated back to 122.95 million yr ago (MYA) (Simillion et al., 2002), placing the duplication event at the root of the *Pentapetalae* clade, which started to diverge between 110 to 124 MYA (Kumar et al., 2017) (Figure 4c).

**FIGURE 5:**
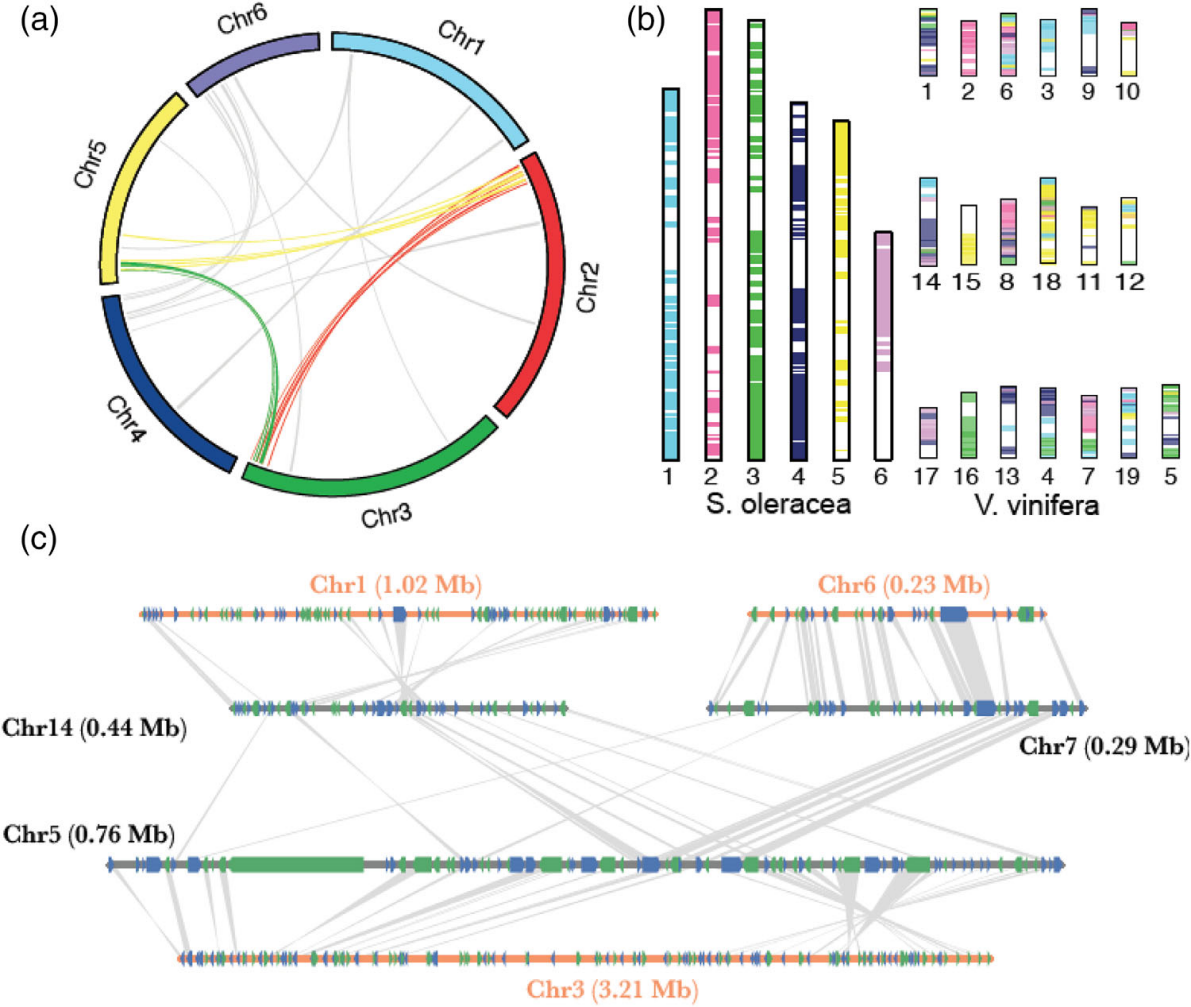
Reconstruction of the paleohexaploid event in spinach based on collinearity between spinach and grapevine genomes. (a) Circle plot comparing syntenic gene blocks within the spinach genome Spov3. (b) The length of the chromosomes is proportional to the assembled chromosome length. The grapevine chromosomes were grouped into three chromosomes per column except for chromosome 5, with each column designated to approximately one of the ancestral protochromosomes (Jaillon et al., 2007). (c) Microsynteny between spinach chromosomes 1, 3, and 6 and a set of ancestral protochromosomes in grapevine. Chromosomes colored in orange are spinach and grey‐colored are grapevine. The values in the parentheses are the lengths of the chromosome section in megabase pairs. The chromosome sections were not drawn proportional to their lengths. Each box on the chromosome axes represents one gene, while blue colored genes are on the forward strands, and green colored genes from the reverse strands

Despite that only 1.5% of the spinach genes were found as anchor genes across the collinear regions, as compared with Arabidopsis (11.8%), grapevine (15.03%), and quinoa (53.65%), the collinearity among chromosomes 2, 3, and 5 suggested that a whole‐genome triplication in spinach could be the gamma triplication event (Figure 5a). We mapped the Spov3 genome to the grapevine chromosomes with 24% of annotated spinach genes anchored in collinear blocks with the grapevine genome. The patterns on grapevine chromosomes coded by spinach chromosomes indicated that the ancestral protochromosomes do have three distinctive copies in spinach; however, they have broken down, fused together and rearranged in the spinach genome (Figure 5b). The triplicate collinear blocks in spinach chromosomes 2, 3, and 5 were mapped to one protochromosomes group on grapevine chromosomes 2, 15, and 16, respectively (Figure 5b).

The divergence between orthologs and the extensive chromosomal rearrangement has resulted in very few collinear blocks identifiable in spinach. By using the collinearity with the grapevine genome, we were able to thread together spinach genome triplicates originating from the paleohexaploidization. The few collinear blocks found between chromosomes 4 and 6 were reconstructed into another ancestral protochromosome consisting of grapevine chromosomes 1, 14, and 17. The data suggest that regions on spinach chromosomes 1, 3, and 6 evolved from one ancestral region represented by grapevine chromosome 14, 5, and 7, respectively, even though no direct collinearity was found among them (Figure 5c). While the region in spinach chromosome 6 almost preserved the ancestral arrangement, large insertions and inversions were observed within the corresponding regions on spinach chromosomes 1 and 3, resulting in a much longer stretch on these chromosomes.

## 4 DISCUSSION

We constructed and compared two independent genome sequences for the spinach cultivar Viroflay and a previously published genome Spov1 (Xu et al., 2017), showing significant improvement of long‐read sequencing platforms over short‐read platforms (Figure 1a). Long‐read platforms not only improve the assembly, but allow for anchoring and orientation of scaffolds, essential for genomic‐assisted breeding (quantitative trait loci and genome‐wide association study), gene discovery using fine mapping, and analysis of synteny across genomes. The two short‐read genomes have similar statistics in assembly and annotation for number of genes (25,495 Spov1 vs 26,862 Spov2). Both of our genomes (Spov2 and Spov3) were annotated with the same transcriptomes derived from a combination of RNA sequencing and Iso‐Seq using the same annotation pipeline. Despite this, the results are drastically different with Spov3 having 8,015 more genes predicted than Spov2 although the sequence of all but 50 PacBio gene models were found in Spov2. Both genome assemblies yielded models unique to their assemblies with common gene models having slightly longer coding sequences (Figure 2b). Although the gene model size is only slightly larger in Spov3, the functional annotation is much improved as indicated by BUSCO scores, the high (92.5%) functional annotation, the number of transcription factors (3,702 vs. 1,202) predicted in Spov3, and an additional 865 unreported resistance genes (1,004 vs. 139) compared with Spov1 (Xu et al., 2017). We further verified that the protein coding genes by checking for presence of transposon without the annotation resulting in 33,660 predicted protein coding genes in Spov3. Overall, compared with the published spinach genome Spov1, the Spov3 genome represents an over 108‐fold increase in contiguity at the contig level, over 42% (328 Mb) higher fraction of the sequence anchored at the chromosome level, and has over 9,300 newly predicted genes (relative to Spov1), all important features for a genome assembly to advance genetic studies in spinach.

Our genotypic and phenotypic analyses of genetic diversity using a subset of the USDA germplasm collection cluster germplasm in spinach leaf types, but more detailed analyses in, for example, leaf texture and type, indicate that, as expected, because of their dioecious nature, spinach collection accessions represent populations. Leaf texture was consistent in only 19 out of 65 (29.2%) accessions between our study and Hayes et al. (2020; Supplemental Table S19) and in 27 of the 66 (40.9%) lines for leaf shape. For this reason, we focused on the leaf type in this study, as the differences can be visualized when the PCA plots with leaf type labels were compared between studies (Supplemental Figure S3). Previous association studies with expanded sets from the USDA spinach collection have yielded mixed results with low proportion of phenotypic variance explained for traits even with high heritabilities (Awika et al., 2019; Qin et al., 2017; Shi et al., 2016), likely as a result of heterogeneity of genotypes within accessions or high population structure. Selection and selfing or sib‐mating within each accession or studying subsets of populations is necessary to refine population and association analyses.

The improved long‐read assembly sheds light on a growing body of evidence for evolution and divergence of species, families and orders in flowering plants. Jaillon et al. (2007) defined an ancestral eudicot genome with *n* = 7 chromosomes based on the grapevine genome, a basal Rosid I. The authors also suggested that a whole‐genome triplication event, named γ, characterizes core eudicots making them paleohexaploids. Since then, paleohexaploidy and its timing has been verified in several species in both eurosids (Myburg et al., 2014) and euasterids (Reyes‐Chin‐Wo et al., 2017; Sato et al., 2012). In general, Rosid 1 (*Populus* and *Vitis*) show large blocks of synteny with the ancestral eudicot genome with more dissection and additional WGDs in *Brassicales* (Jaillon et al., 2007). Conversely, the euasterids are characterized by several additional WGD with gene rearrangements being the norm. For example, WGDs are detected in *Ericales* (Larson et al., 2020) with independent WGDs for Asterid I (Sato et al., 2012) and even within Asterid II between *Lactuca* (Reyes‐Chin‐Wo et al., 2017), *Helianthus* (Badouin et al., 2017), and *Daucus* (Iorizzo et al., 2016). Reyes‐Chin‐Wo et al. (2017) suggested that perenniality and generation time affected divergence of plant species.

Evolutionary time of an ancient WGD event can be estimated using *Ks* values in paralogs that are anchored in collinear blocks (Tiley et al., 2018). This strategy helped to date the paleohexaploidization using the few collinear blocks preserved in the spinach genome. The gamma triplication event was dated in Arabidopsis to about 156 MYA. Other targeted studies on eudicots estimate the triplications around 120 ± 2.05 MYA (Vekemans et al., 2012). Duplication events happened in the evolution history could go undetected because of chromosomal rearrangement, difficulty to tease apart multiple duplication events, variable evolutionary rates, or lack of high‐quality genome resources. We confirm that spinach is a paleohexaploid with no further WGDs, as reported in previously (Xu et al., 2017) and for other members of Caryophyllus (Dohm et al., 2012). With the highly contiguous and genetically verified assembly afforded by long‐reads in spinach, we show for the first time that despite substantial gene rearrangements unreported remnants of paleohexaploidy can be further detailed by using bridge species such as grapevine. As no additional recent WGD was detected in spinach, the gamma triplication residues were not masked by the overwhelming amount of younger rearrangements first reported in this paper (Figure 5a). The use of a pivotal genome such as grapevine helps to elucidate the ancient duplication events in a genome (Abrouk et al., 2010; Wang et al., 2018). As an increasing number of species’ genomes are being developed using long‐read and scaffolding technologies, chromosomal‐level, highly contiguous assemblies will allow further definition of speciation at the whole‐genome level and importantly at the gene family level. This is particularly important for identification of copy number variants to understand and manipulate gene function (Alonge et al., 2020).

## CONFLICT OF INTEREST

The authors declare no conflict of interest

## AUTHOR CONTRIBUTIONS

AVD conceived the project. KS collected, extracted and processed the plant samples. JC, MS, TG performed the genome assembly. AV, AMHK, HB and MI led analyses. AMHK, HB, SC, HA, LL, SC, ET, WS, MI performed data analysis. AMHK, HB, SC, AVD wrote the paper. All authors participated in discussions, provided valuable advice, and read and approved the final version of the manuscript.

## Supporting information

Supplemental Material

Supplemental Figure S1. K‐mergenome size estimate from Illumina Sequence Data.
Supplemental Figure S2. Venn Diagram of functional annotation for SpoV3
Supplemental Figure S3. Principal Component Analysis comparison labeled by leaf type. A.) Phenotypes labeled as in main text derived from phenotyping at University of California, Davis. B.) Phenotypes derived from Hayes et al. (2020).
Supplemental Figure S4. Visualization of Spinach Genetic Linkage Map. Genetic map was developed with skim resequencing of 77 recombinant inbred lines at the F6 generation using MSTMapand plotted using MapChartsoftware.
Supplemental Figure S5. Mummerplot comparison of Spinach Assembly Versions Spov1 and Spov3. Alignments are plotted for shared alignments greater than 5,000 base pairs.

Supplemental Table S1: Summary of sequence libraries generated through Illumina whole genome sequencing on the Hiseq 2000 of *S. oleracea*variety Viroflay following quality filtering.
Supplemental Table S2: Estimation of *S. oleracea* genome size based on K‐mer analysis.
Supplemental Table S3: Assembly statistics for scaffolds and scaftigs of *Spinacia oleracea* and *Beta vulgaris* sequences.
Supplemental Table S4: High‐density spinach genetic linkage map produced by skim resequencing of 77 individuals.
Supplemental Table S5. Genotyping data from NIL Population used for Linkage Mapping.
Supplemental Table 6S. RNA sequencing samples utilized for annotation. All samples were used to produce Illumina Libraries, those marked with asterisk (*) were also used to generate Pacific Biosciences Iso‐Seq Libraries.
Supplemental Table S7: BUSCO v4 conserved ortholog analysis results for Spov3 with core Eudicotyledons gene set.
Supplemental Table S8: Status of COGENT reconstructed Pacific Biosciences Iso‐Seq gene families presence in Pacific Biosciences and Illumina de novo genome assemblies.
Supplemental Table S9: List of COGENT identified Spov2 contigs containing gene families that were not present and therefore added to the Spov3 assembly.
Supplemental Table S10. Repetitive content summary of the Spov3 genome assembly using RepeatMasker.
Supplemental Table S11: Summary statistics of gene models in Spov2, Spov3 compared to some other model organisms and close relative species.
Supplemental Table S12. Summary of the functional annotation for the Spov3 genes.
Supplemental Table S13. List of genes in Spov3 that are transposable elements
Supplemental Table S14. List of genes in Spov3 that are transposable elements
Supplemental Table S15. Comparison of transcription factors between spinach assemblies
Supplemental Table S16. Summary of Transcription factors identified in Spinacia oleracea genome, Spov3.
Supplemental Table S17. Transcription factors identified in Spinacia oleracea genome, Spov3.
Supplemental Table S18. Summary of resistant genes identified in Spinacia oleracea genomes.
Supplemental Table S19. Resistance genes identified in *Spinacia oleracea* genome, Spov3.
Supplemental Table S20: Resequenced spinach lines and assess leaf characteristic phenotypes.

Supplemental Material

## Data Availability

Raw sequencing reads have been deposited in the NCBI SRA database under BioProject PRJNA663886 and BioSample accession number SAMN06345840, which includes the Illumina and Pacific Biosciences whole‐genome sequences as well as all RNA sequencing data used for annotation. All other supportive sequencing has been uploaded under BioProject PRJNA661027. The final PacBio genome assembly version described in this paper, *Spinacia oleracea* Spov3, is available through Phytozome Database (https://phytozome‐next.jgi.doe.gov/ and https://doi.org/10.5281/zenodo.4623865). The final Illumina genome assembly version described in this paper, *Spinacia oleracea* Spov2, and variant call format file of SNPs from 75 resequenced lines are available from GitHub (https://github.com/USDA‐ARS‐GBRU/Spinach_Peffusa).
