# Supplementary material for: An anchored chromosome‐scale genome assembly of spinach improves annotation and reveals extensive gene rearrangements in euasterids
Source: Plant Genome. 2021 Jun 10;14(2):e20101. doi: 10.1002/tpg2.20101 (PMC12806983; doi:10.1002/tpg2.20101)
Supplement: Supplementary file 1 [file TPG2-14-e20101-s001.pdf]

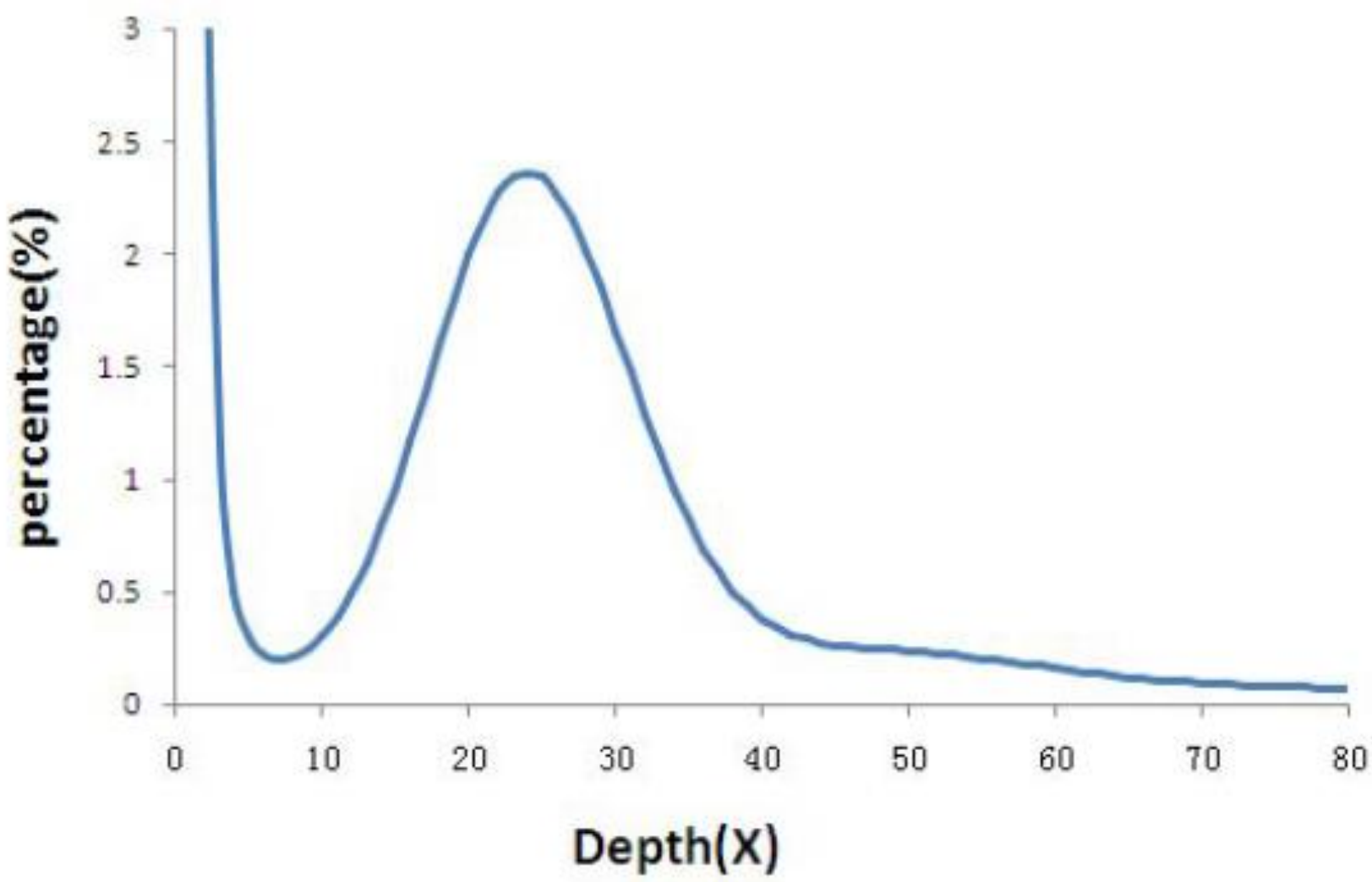

Supplemental Figure 1. K-mer genome size estimate from Illumina Sequence Data. The x-axis is depth (X); the y-axis is the proportion which represents the frequency at that depth divide by the total frequency of all the depth. Without consideration of the sequence error rate, heterozygosis rate and repeat rate of the genome, the 17-mer of distribution should obey the Poisson theoretical distribution. From the actual data, due to the sequence error, the low depth of K-mer frequency will take up a large proportion. At the same time, for some specific genome, the certain heterozygosis rate can cause a sub peak at the position of the half of the main peak, while a certain repeat rate can cause a repeat peak at the position of the integer multiples of the main peak.

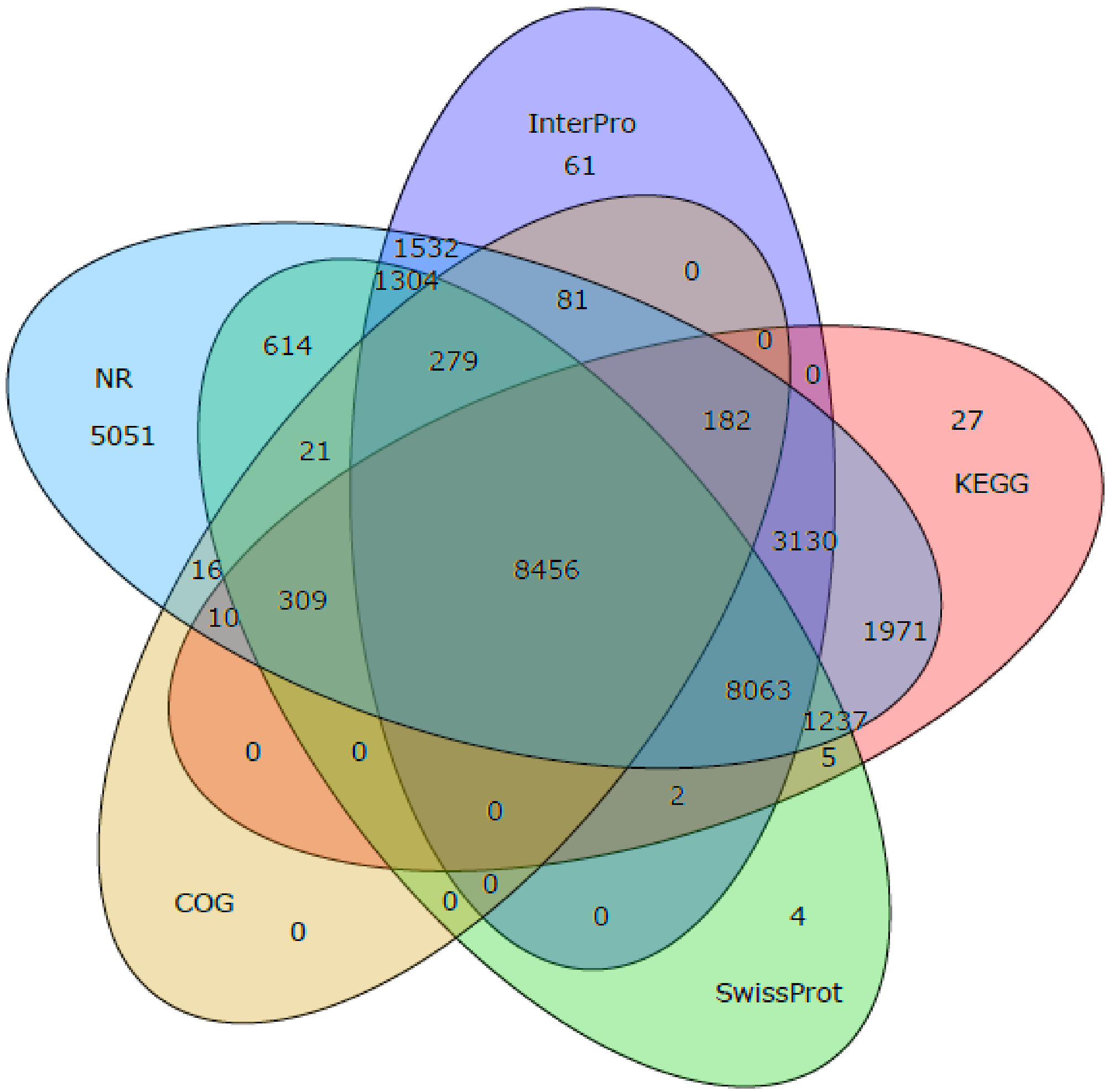

Supplemental Figure S2. Venn Diagram of functional annotation for SpoV3

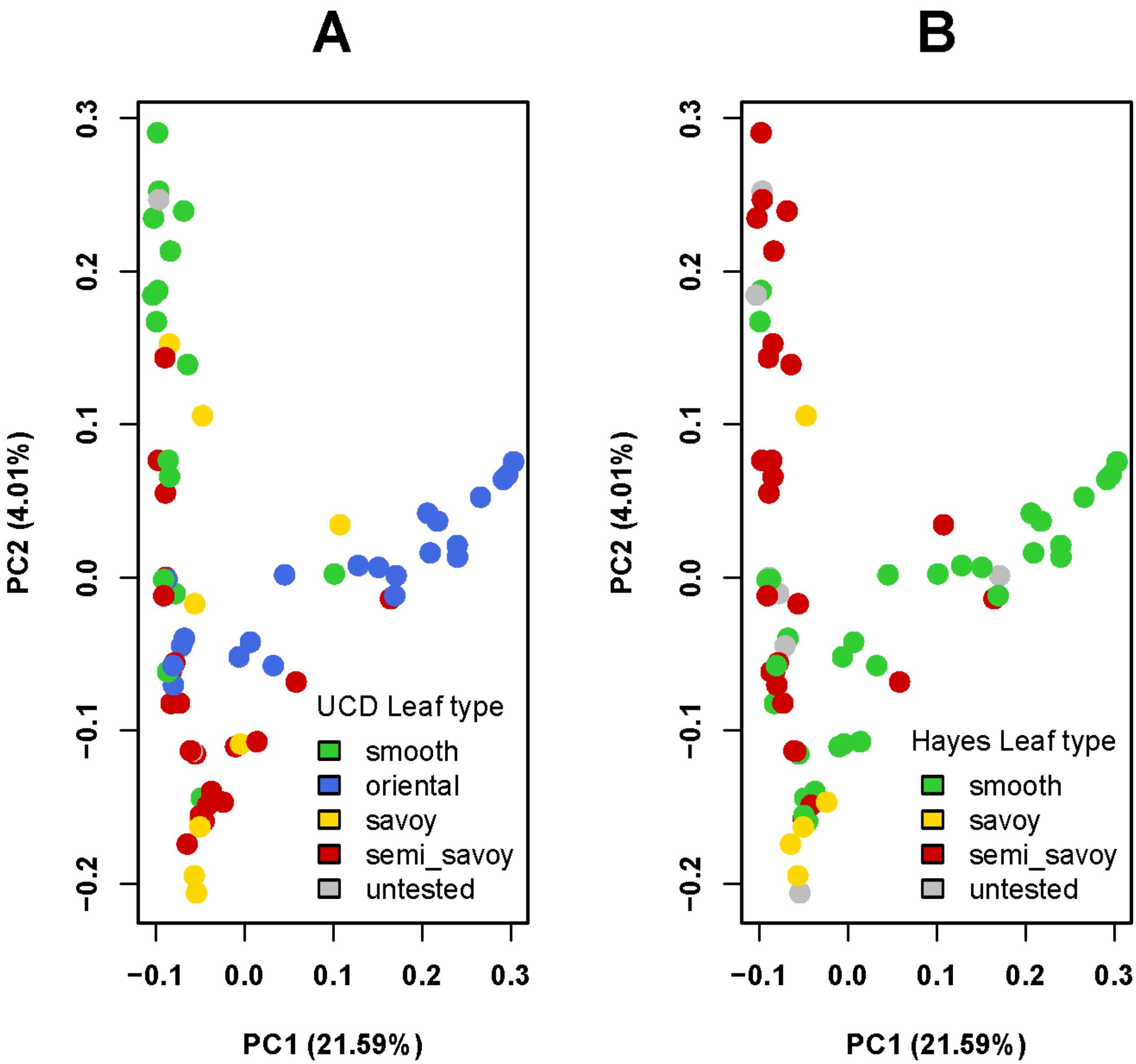

Supplemental Figure 3. Principal Component Analysis comparison labeled by leaf type. A.) Phenotypes labeled as in main text derived from phenotyping at University of California, Davis. B.) Phenotypes derived from Hayes et al. (2020).

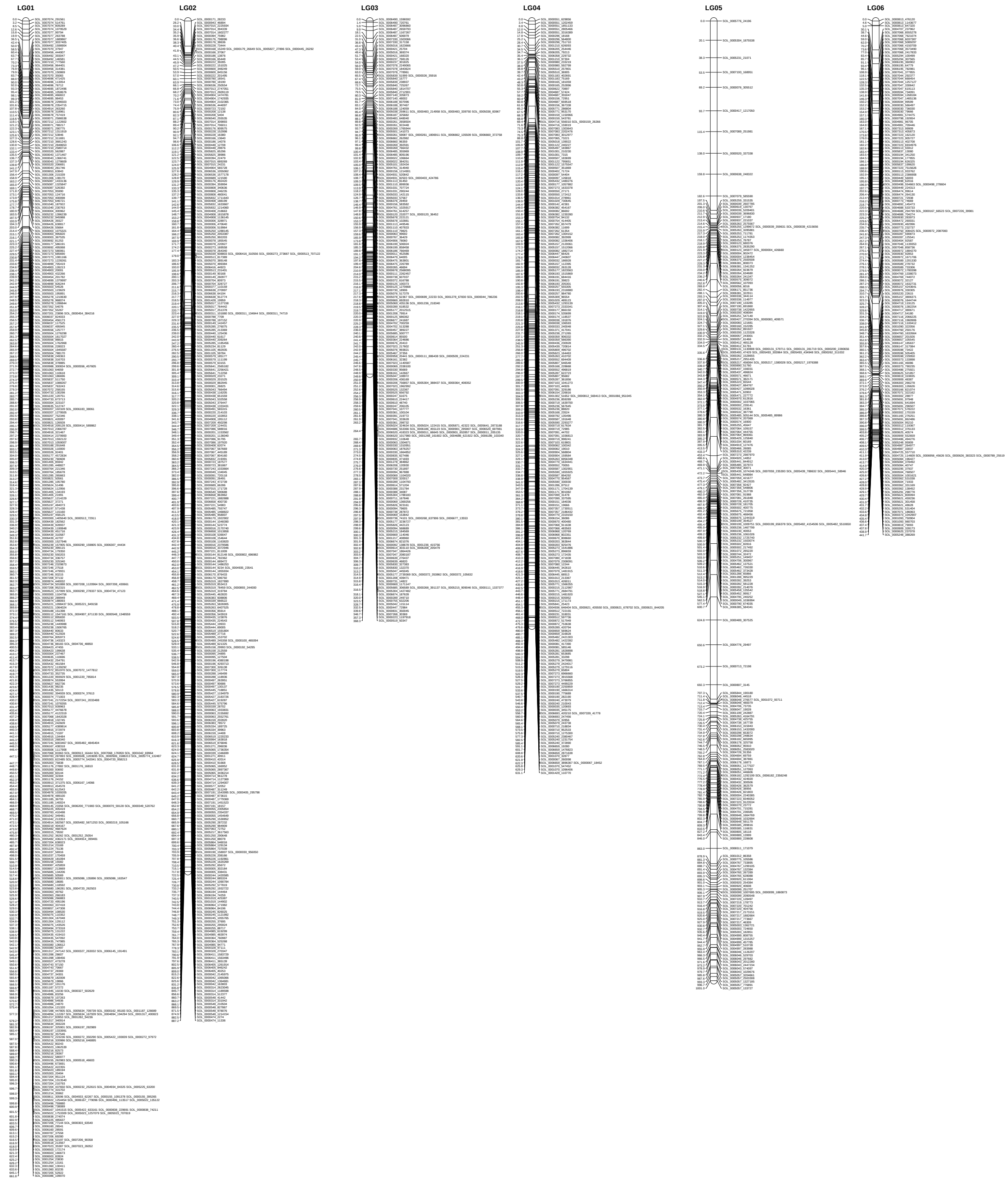

Supplemental Figure 4. Visualization of Spinach Genetic Linkage Map. Genetic map was developed with skim resequencing of 77 recombinant inbred lines at the F6 generation using MSTMap and plotted using MapChart software.

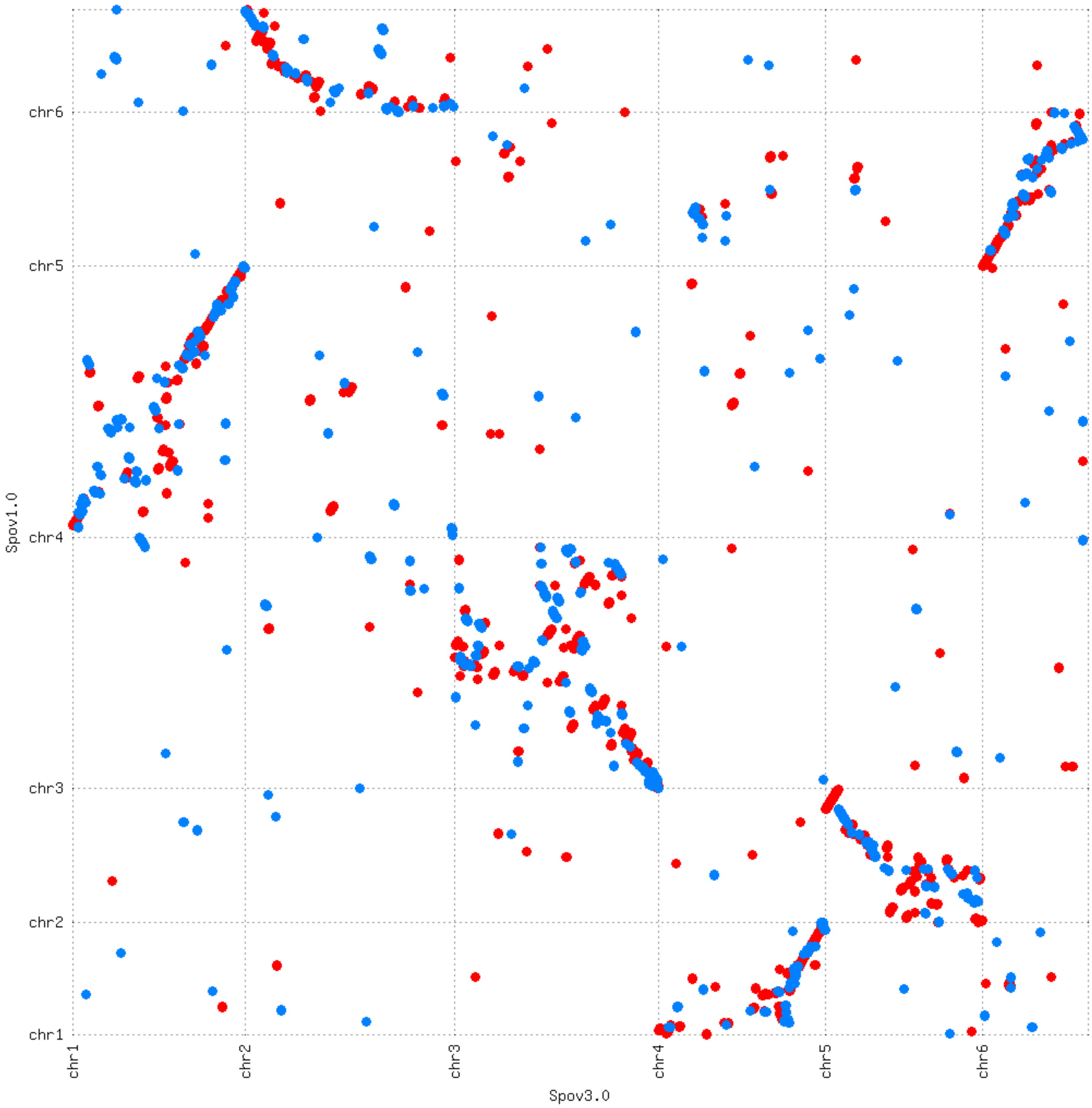

Supplemental Figure 5. Mummerplot comparison of Spinach Assembly Versions Spov1 and Spov3. Alignments are plotted for shared alignments greater than 5,000 base pairs.
