# Supplementary material for: An anchored chromosome‐scale genome assembly of spinach improves annotation and reveals extensive gene rearrangements in euasterids
Source: Plant Genome. 2021 Jun 10;14(2):e20101. doi: 10.1002/tpg2.20101 (PMC12806983; doi:10.1002/tpg2.20101)
Supplement: Supplementary file 3 [file TPG2-14-e20101-s003.docx]

# **Supplemental Materials and Methods**

## ***Plant material and DNA sequencing of variety ‘Viroflay’***

Ninety-four plants homozygous for 384 random SNP markers (data not shown) were selected for sequencing from the monoecious spinach heirloom variety, Viroflay, a large smooth leaf type. Young leaf tissue from these plants grown in the dark for 48 hours were collected in bulk and flash frozen in liquid nitrogen. DNA was isolated using a modified CetylTrimethyl Ammonium Bromide (CTAB) extraction, detailed in Stoffel et al. (Stoffel et al., 2012). The quality and size range of the isolated DNA was confirmed by gel electrophoresis (0.3% agarose) for 24h at 30V. DNA was quantified by Picogreen and the DNA purity was verified by NanoDrop Spectrophotometer (Thermo Fisher Scientific, Germantown, USA). The sample was partitioned for sequencing with both Illumina and Pacific Biosciences technologies.

Genomic DNA was sheared and size-selected to generate eight Illumina paired-end libraries to sequence the Viroflay genome (**Supplemental Table 1)**. Libraries were constructed at BGI (Shenzhen, China) according to the manufacturer’s instructions and paired-end (PE) sequencing (2x100bp) was performed on the Illumina HiSeq 2000. These included three paired-end libraries with insert sizes of 170, 250 and 500 bp and five mate-pair (MPE) libraries with insert sizes of 2, 5, 10, 20 and 40 kb. The quality of each library was validated using Qubit®, AGE.

The same genomic DNA as specified above was utilized for Pacific Biosciences sequencing. It was sheared to obtain fragments of 20-25Kb using Covaris g-tubes and prepared into SMRTbell libraries using the SMRTbell Template Prep kit 1.0 according to manufacturer’s instructions (Pacific Biosciences, Menlo Park, USA). Libraries were size-selected using the Blue Pippen system with 15 and 20Kb cutoff. SMRTBells were sequenced with the P3C3 chemistry and 180-minute movie times on the Pacific Biosciences RSII. Hereafter we will refer to these sequences as PB.

## ***RNA extraction, library preparation, and sequencing***

Total RNA was extracted from leaf samples using the RNeasy Mini Kit (Qiagen, Valencia, USA) according to manufacturer’s instructions of 100mg of tissue for each sample, and yields were quantified using a Qubit 2.0 Fluorometer (Invitrogen, Carlsbad, USA). Libraries from each tissue type were constructed and sequenced on an Illumina HiSeq 4000 (Illumina, Carlsbad, USA) at the DNA Technologies and Expression Analysis Core Laboratory, University of California Davis to generate paired-end 150 reads from all samples (**Supplemental Table 6**). SFI, RFI, FSVI and FRVI samples were also utilized to generate Pacific Biosciences IsoSeq libraries which were size-selected for two size fractions containing 1-2kb fragments and 2-6kb fragments. Full-length transcripts were sequenced on the RSII. Hereafter we will refer to these sequences as RNA-seq and IsoSeq for the Illumina RNA sequencing samples and the Pacific Biosciences IsoSeq samples, respectively.

## ***Genome assembly, scaffolding and anchoring***

### **Illumina genome assembly**

Low quality reads were filtered for >10 ‘N’ ambiguous nucleotides, >30 nucleotides with quality scores ≤30, contained adapter sequences, and identical reads/duplicates using an in-house program. A portion (27.6Gb) of the PE filtered reads were used to estimate genome size using the k-mer method with the Jellyfish software. Quality filtered PE data was assembled using SOAPdenovo version 2.04 (Li et al., 2008). To simplify both assemblies and reduce computational complexity, SOAPdenovo employs the de Bruijn graph algorithm. The SOAPdenovo assembly was divided into three steps. First, contigs are constructed by splitting the short-insert-size (170, 250 and 500 nt) PE library data into k-mers and constructing de Bruijn graphs. These graphs are then simplified by removing low-coverage edges and low-coverage k-mers and merging bubbles to produce contigs. Second, scaffolds are constructed by realigning all of the usable paired-end reads onto the contig sequences and calculating the shared paired-end relationships between each pair of contigs. Scaffolds are then constructed by weighing the rate of consistent and conflicting paired-ends. Finally, gaps are filled using GapCloser for SOAPdenovo^4^ which uses the paired-end information to retrieve the read pairs in which one end is mapped to the unique contig and the other is located within a gap region.

### **PacBio genome assembly and scaffolding**

The PB raw sequences were utilized for *de novo* genome assembly using HGAP and Celera assembler software to produce the initial Spinach PacBio assembly. Following the optimizations we previously developed for the Asian Seabass genome (Vij et al., 2016), the HGAP error-corrected reads were assembled using the Celera assembler with the following parameters: frgMinLen = 1000, ovlErrorRate = 0.03, ovlMinLen=200, utgGenomeSize = 989000000, utgErrorRate=0.01, utgGraphErrorRate = 0.01, unitigger=bogart, stopAfter=unitigger. In addition, the unitigging parameters for BOGART were: -B 16032 -eg 0.02 -Eg 3.25 -em 0.045 -Em 5.25 -repeatdetect 10 20 15. The assembled contigs were then polished by Quiver (Chin et al., 2013) using the PacBio raw read sequences, and pilon (Walker et al., 2014), using Illumina PE whole-genome sequence data correcting for small indels and incorrect bases. For scaffolding, indentation and correction of chimeric contigs of this assembly, the three largest MPE (10kb, 20kb, and 40kb) were aligned to contigs utilizing the BWA (Li and Durbin, 2010) software. Uniquely mapped pairs were retained and observed for mapping continuity across scaffolds and where coverage gaps were present. Contigs with numerous pairs supporting mapping across different scaffolds was used for scaffolding contigs. Contig regions with abnormal read coverage (too high or too low) with numerous reads on each side with the other pair mapped on other contigs were further investigated for detection and correction of chimeric regions. PBJelly (English et al., 2012) was used with raw PacBio reads for gap filling.

In order to name the pseudomolecules according to chromosome identification, the SO4* sex-linked simple sequence repeat marker primer sequences were aligned with BLAST to the final scaffolds with default parameters. Primer hits were analyzed to assess where both forward and reverse primers aligned complimentary which would allow for production of a PCR product. The pseudomolecule which contained this location was named as Chromosome 1 based on historical identification of this chromosome in cytological work in spinach. The remaining five main pseudomolecules were named based on descending scaffold lengths (Chr 2-6). Unanchored scaffolds and contigs were named in descending length order.

## ***Comparative analysis between PacBio and Illumina assemblies***

The COGENT software was utilized to analyze the RNA IsoSeq data to *de novo* reconstruct the transcribed regions of each gene by using full-length transcript sequences (Workman et al., 2018). These are then partitioned into gene families. The results of these gene family partitions were compared using CD-HIT-EST. As results were determined to be more precise from COGENT, these gene families were utilized in downstream analysis to investigate completeness of the gene space in the Illumina and PacBio genome contig assemblies by aligning the gene family using minimap (Li, 2016). Any contigs that were found to contain gene families in the Illumina assembly that were missing in the PacBio assembly were extracted and added to the PacBio assembly.

## ***Resequencing, HapMap Construction and Linkage Mapping***

Whole genome paired-end sequencing (WGS) of 8-10X genome equivalents was obtained for spinach lines representing the four phenotypically different spinach types: smooth, oriental, savoy and semi-savoy. A total of 75 lines were included as outlined in **Supplemental Table 20**. Genomic DNA was extracted from young leaf tissue that had been flash frozen in liquid nitrogen and extracted with Qiagen DNAeasy Plant Kit according to manufacturer’s instructions. DNA was tested for quality using Nanodrop Spectrophotometer (Thermo Scientific, Germantown, USA). DNA was sheared using Covaris g-tubes and used to generate Illumina TruSeq Libraries for sequencing on the Illumina HiSeq 3000 to produce 2x150bp reads.

The raw Illumina reads were trimmed for adapter/primer sequences and any overlapping reads were merged using CLC Genomics workbench (V7.0). The first 13 bases of paired reads and 13 bases from each side of merged reads were trimmed. Trimmed reads were mapped to the initial PacBio Genome Assembly. Mappings were exported in BAM format and utilized with SAMtools to call SNPs according to the established pipeline in Hulse-Kemp et al. (2016). To compensate for errors in sequencing, if a base represented >90% of reads, it was considered homozygous. The population SNP file was filtered to extract the final high-confidence set based on missing data <20% at each individual locus position.

A population of 77 recombinant inbred lines at the F6 generation were utilized as a segregating population for genetic mapping. Lines were derived from two breeding lines lines, one representing an oriental type and one representing an early-medium type. DNA from both parents and all 77 lines were obtained using DNeasy Plant Mini Kits (Qiagen GmbH). Libraries for each sample were pooled in equimolar concentrations and 2x150 bp paired end sequencing was run on an Illumina Hiseq 3000. Demultiplexed samples were assessed for quality using fastQC. Individual read files were then quality trimmed and the first 15 bases and last 15 bases were removed due to general bad quality across the majority of the reads in CLC genomics workbench. Reads for each sample were aligned to the Spov3 contigs genome in CLC genomics workbench version 8 utilizing default settings asides from a similarity fraction of 0.9, length fraction 0.6 and requiring a unique alignment. A mapping file for each sample was exported in BAM format for utilization in SNP calling.

Genotypes were called for all mapping population samples at positions that were identified as polymorphic between the parents during the high-coverage resequencing analysis utilizing the HaplotypeCaller of GATK version 3.5. Genotypes that were determined by three or more reads at the locus position were retained then SNP positions were filtered to retain those with minor allele frequency greater than 10% and greater than 40% of the samples having a called genotype. Files were converted to ABH mapping format and imported into JoinMap version 4.1, loci were grouped using the independent LOD algorithm examining groups with LOD greater than 7. Within each identified group, the percentage of missing genotypes and percentage of heterozygous genotypes and their distributions were analyzed. Markers within a contig were ranked based on missing data percentage for the marker and indicated if they contained greater than one standard deviation (SD) from the average heterozygosity percentage within the linkage group. Ranks and positions of markers were utilized to select optimally three or more representative markers for each contig when possible, with a low missing percentage and a non-significantly elevated level of heterozygotes that, and that were spaced 200kb apart. Heterozygous genotypes for all loci with greater than 1 SD of heterozygous were converted to missing. VCF files containing all markers were converted using Stevison lab scripts (vcf2fastPHASE.pl/fastPHASE2vcf.pl) and missing data was imputed using fastPHASE.

Imputed genotypes for the selected markers were converted to ABX mapping format for import into MSTMap (**Supplementary Table 5**), heterozygous genotypes for markers that were the only representative of a sequence contig were converted to missing genotype, due to fastPHASE imputing all missing genotypes in the contig as heterozygotes. Files were imported into MSTMap for each linkage group separately and ordered based on markers belonging to a single group (RI6 population type and kosambi mapping function). MSTMap was run two times: 1) with all samples and 2) removing two low depth samples based on initial resequencing data for the samples (110 and 119). Map positions of markers that are located on the same sequence contig were analyzed for both methods, in order to calculate the percentage of markers that integrated into adjacent positions in the linkage maps as well as when markers integrated nearby but with markers (s) integrated that separated the markers from ordering into a single adjacent group from only a single other sequence contig. The order that obtained the highest percentage of adjacently ordered markers for contigs with two or more markers was selected as the final order for each linkage group (**Supplementary Table 4**). Linkage groups were drawn with MapChart version 2.3 (**Supplementary Figure 4**).

## **Phenotyping and population analysis of diversity materials**

At UC Davis, 4-6 plants from each of 75 lines were grown in 4-inch pots in the greenhouse at 24/18 ⁰C day/night temperatures with no additional lighting in spring 2017. Phenotypic data on leaf type was collected at five weeks after planting. Leaf type was classified as smooth, semi-savoy, savoy and oriental (arrowhead).

The SNPs detected in the 75 resequenced lines were further filtered for minor allele frequency >0.05 and then missing rate was evaluated on a per individual basis. Individuals with >20% missing data were excluded from further analysis. The variant call format file was used in plink version 1.9 to perform principal coordinate analysis and extract eigenvectors which were plotted using R version 3.6.1. Samples were labelled using phenotypic information gathered as mentioned above as well as from the data referenced in Hayes et al. (Hayes et al., 2020). The VCF file was also formatted for fastStructure version 1.0 using plink version 1.9. The function of chooseK.py was tested for K=2-10 for the population using fastStructure and a final K of 3 was utilized for further analysis. Structure calculated from K=3 was plotted using the distruct.py algorithm. Dendrograms for the population were calculated using the R package ‘SNPRelate’.

## **Construction of final pseudomolecules**

Chromosome pseudomolecules were constructed from the assembly contigs based on the skim resequencing derived linkage map. The length of final pseudomolecules was calculated and used to assign chromosome numbers based on length of scaffolds in descending order. In order to identify the sex-linked chromosome which has traditionally been referenced via *in situ* hybridization studies as chromosome 1 in spinach. A sex-linked simple sequence repeat marker, SO4* (Khattak et al., 2006) as aligned to the pseudomolecules with default parameters using BLAST. Hits for forward and reverse primer locations were analyzed to determine the one feasible genomic location producing the amplification result with these primers, this location was used to label the corresponding pseudomolecule as Spov3_chr1. The nomenclature for sequences is Spov3_Chr# for the six chromosome pseudomolecules, in which Spo indicates the *Spinach oleracea* genome. Spov3 is also the NCBI registered locus tag prefix for gene annotations, v3 represents the final version of the genome assembly, and “#’ is the unique numeric identifier for the sequence. Spov3 genome assembly is the final genome version utilized in all downstream analyses.

To identify the correlation to the previously published draft Spinach genome version 1 (Xu et al., 2017) with the Spov3 genome assembly, the developed pseudomolecules were aligned against the 6 Spov1 chromosomes using Mummer software (version 4) considering the parameters: (-mum –c 5000). The resulting alignments is plotted using mummerplot (**Supplementary Figure 5**).

***Repeat analysis***

Repeat analysis was done in a two step process. Initially, the Spov3 assembly sequences were used as input to RepeatModeler a *de novo* repeats identification and annotation pipeline to identify species specific repetitive elements, using the NCBI engine and the generated database of Spov3 from BuildDatabase module implemented in the RepeatModeler. RepeatMasker a program to screens DNA sequences for interspersed repeats and low complexity DNA sequences, was then used to annotate and mask the genome sequence using a custom library (a hybrid library generated from RepeatModeler and plant section of the Repbase repeats database) using the following parameters –pa 30 -gff -norna -no_is –x -lib. The resulted annotation file and masked genome were later used for the gene prediction and downstream analyses.

## ***Gene prediction and annotation***

*De novo* prediction was carried out following the methods outlined in Li et al. (Li et al., 2020). Briefly, AUGUSTUS v2.5.5 (Stanke et al., 2006) was used to train gene models assembled by Bridger utilizing all RNA sequencing data sets (**Supplemental Table 6)** which were quality trimmed for low quality bases and adapter content using fastx toolkit (Gordon and Hannon, 2010). All programs were trained on training sets from the model species *A. thaliana* and *S. lycopersicum*. The protein sequences of *S. lycoperisum* v2.3, *S. tuberosum v3.4*, Vitis vinifera (Jaillon et al., 2007), *A. thaliana*, *B. vulgaris (Dohm et al., 2014)*, and *D. carota (Myburg et al., 2014)* were mapped to the spinach genome using tblastn^11^ (Blastall 2.2.23 ) to generate protein-based gene models. Only hits with an E-value <1e-5 were retained and further analyzed with GeneWise 2.2.0 (Birney et al., 2004) to search the most accurate spliced alignments. All gene models produced by *de novo* prediction and protein-homology searches, were integrated using MAKER (v.2.31.8) (Cantarel et al., 2008). Summary statistics of the gene models predicted for Illumina and PacBio genomes as well as 4 other high-quality and reference plant genome species of *A. thaliana*, *B. vulgaris* (Funk et al., 2018), *S. lycopersicum* , and *D. carota (Iorizzo et al., 2016)* were generated using an in-house script. This was used to compare the structural properties of gene sets for identifying any abnormalities.

Putative gene functions were assigned according to the best match of the alignments using BLASTp^11^ (E-value≤10x10^-5^) to SwissProt and TrEMBL databases. The motifs and domains of genes were determined by InterProScan version 4.7 (Zdobnov and Apweiler, 2001) against protein databases including ProDom, PRINTS, Pfam, SMART, PANTHER and PROSITE. Gene Ontology IDs for each gene were obtained from the corresponding InterPro entries. All genes were aligned against KEGG (Release 58) proteins, and the pathway in which the gene might be involved was derived from the matched genes in KEGG. Gene models with no match in these databases were labeled “hypothetical proteins.”

### **Comparative analysis between PacBio and Illumina gene prediction**

All PacBio genome predicted gene models were transferred to the Illumina genome assembly using GMAP (Wu et al., 2016) a standalone program for mapping and aligning cDNA sequences to a genome using the –f 2 parameter. The transferred gene models from PacBio genome were then intersected with the Illumina predicted gene models on the same genome using an in-house script, and different categories of exact match, partial match and no match were flagged for each gene model from the corresponding set. Several stats for each category such as number of models, locus, CDS and intron length, and average number of CDS and introns were calculated for further exploration and investigation.

### **Transcription factors and resistance genes**

PlantTFcat (Dai et al., 2013) a reference plant transcription factor and transcriptional regulator categorization tool was used to predict all the transcription factors and regulatory genes in Spov3 gene models. PRGdb 3.0 (Osuna-Cruz et al., 2018), a comprehensive platform for prediction and analysis of plant disease resistance genes was used to predict the disease resistant genes in the Spov3 gene prediction collection. The tool Matrix-R automatically retrieves, annotates and classifies plant resistance genes. Protein sequences of 91 to date cloned R-genes were downloaded from PRGdb and aligned using MUSCLE 3.6 (Edgar, 2004) with manual editing. The resulting alignments for each R-class were used as a base for the creation of an aligned subset of conserved regions and of a set of hidden Markov models (HMMs). A total of 60 HMM were build, 15 for the CNL class, 24 for the TNL class, 8 for the RLK class and 13 for the RLP class. The input dataset of Matrix-R is a plant proteome. For each protein, Matrix-R calculates: the coils potential, to detect CC domains, the putative Transmembrane to infer the putative protein localization, the matching score with the previously created HMM modules.

### **Collinearity analysis between spinach and other genomes**

Four high-quality eudicot genomes, including arabidopsis v11, grape (Jaillon et al., 2007), sugar beet (Funk et al., 2018), quinoa (Jarvis et al., 2017), were downloaded from their respective databases. Protein sequences were aligned within the genomes and against spinach with default setting in BLAST. The collinear blocks were identified using MCScanX (Wang et al., 2012) with at least 5 anchor gene pairs and fewer than 25 genes in between the anchor genes. Ks values were calculated in the anchor gene pairs by aligning the coding sequences using MUSCLE and using Nei-Gojobori statistics implemented in MCScanX. The distributions of Ks values were plotted in R (R Development Core Team). Estimated age of WGD was calculated by Ks/ (2λ) with λ the rate of synonymous substitution in arabidopsis equal to 6.1x10^-9^ (Simillion et al., 2002). A species tree was build in OrthoFinder using whole genome protein sets from all five eudicot genomes plus rice and sorghum (Cooper et al., 2019; Kawahara et al., 2013). The branch length of the species tree was adjusted to evolution time using the TimeTree database (Kumar et al., 2017). The circle plot of collinear blocks in spinach genome was generated using Circos (Krzywinski et al., 2009). The microsynteny plot was generate using JCVI python tools for MCScan (Tang et al., 2015).

# **References**

RepeatMasker version 4.0.7. [Online] <http://www.repeatmasker.org/> (verified March 15).

Solanaceae Genomics Network [Online] <https://solgenomics.net/organism/Solanum_lycopersicum/genome> (verified June 10).

The Arabidopsis Information Resource (TAIR) [Online] <https://www.arabidopsis.org/download/index-auto.jsp?dir=/download_files/Genes> (verified October 11).

Birney, E., M. Clamp, and R. Durbin. 2004. GeneWise and genomewise. Genome research 14:988-995.

Cantarel, B.L., I. Korf, S.M.C. Robb, G. Parra, E. Ross, B. Moore, C. Holt, A. Sánchez Alvarado, and M. Yandell. 2008. MAKER: An easy-to-use annotation pipeline designed for emerging model organism genomes. Genome Res 18:188-196, <http://genome.cshlp.org/content/18/1/188.abstract>.

Chin, C.-S., D.H. Alexander, P. Marks, A.A. Klammer, J. Drake, C. Heiner, A. Clum, A. Copeland, J. Huddleston, and E.E. Eichler. 2013. Nonhybrid, finished microbial genome assemblies from long-read SMRT sequencing data. Nature methods 10:563-569.

Cooper, E.A., Z.W. Brenton, B.S. Flinn, J. Jenkins, S. Shu, D. Flowers, F. Luo, Y. Wang, P. Xia, and K. Barry. 2019. A new reference genome for Sorghum bicolor reveals high levels of sequence similarity between sweet and grain genotypes: implications for the genetics of sugar metabolism. BMC genomics 20:1-13.

Dai, X., S. Sinharoy, M. Udvardi, and P.X. Zhao. 2013. PlantTFcat: an online plant transcription factor and transcriptional regulator categorization and analysis tool. BMC bioinformatics 14:1-6.

Dohm, J.C., A.E. Minoche, D. Holtgräwe, S. Capella-Gutiérrez, F. Zakrzewski, H. Tafer, O. Rupp, T.R. Sörensen, R. Stracke, R. Reinhardt, A. Goesmann, T. Kraft, B. Schulz, P.F. Stadler, T. Schmidt, T. Gabaldón, H. Lehrach, B. Weisshaar, and H. Himmelbauer. 2014. The genome of the recently domesticated crop plant sugar beet (Beta vulgaris). Nature 505:546-549, <https://doi.org/10.1038/nature12817>.

Edgar, R.C. 2004. MUSCLE: multiple sequence alignment with high accuracy and high throughput. Nucleic acids research 32:1792-1797.

English, A.C., S. Richards, Y. Han, M. Wang, V. Vee, J. Qu, X. Qin, D.M. Muzny, J.G. Reid, K.C. Worley, and R.A. Gibbs. 2012. Mind the Gap: Upgrading Genomes with Pacific Biosciences RS Long-Read Sequencing Technology. PLOS ONE 7:e47768, <https://doi.org/10.1371/journal.pone.0047768>.

Funk, A., P. Galewski, and J.M. McGrath. 2018. Nucleotide‐binding resistance gene signatures in sugar beet, insights from a new reference genome. The Plant Journal 95:659-671.

Gordon, A., and G. Hannon. 2010. Fastx-toolkit. FASTQ/A short-reads preprocessing tools (unpublished) <http://hannonlab>. cshl. edu/fastx_toolkit 5.

Hayes, M., M. Pottorff, C. Kay, A. Van Deynze, J. Osorio-Marin, M.A. Lila, M. Iorrizo, and M.G. Ferruzzi. 2020. In Vitro Bioaccessibility of Carotenoids and Chlorophylls in a Diverse Collection of Spinach Accessions and Commercial Cultivars. Journal of Agricultural and Food Chemistry 68:3495-3505, <https://doi.org/10.1021/acs.jafc.0c00158>.

Hulse-Kemp, A.M., H. Ashrafi, J. Plieske, J. Lemm, K. Stoffel, T. Hill, H. Luerssen, C.L. Pethiyagoda, C.T. Lawley, M.W. Ganal, and A. Van Deynze. 2016. A HapMap leads to a Capsicum annuum SNP infinium array: a new tool for pepper breeding. Horticulture Research 3:10, <Go to ISI>://WOS:000381195100001.

Iorizzo, M., S. Ellison, D. Senalik, P. Zeng, P. Satapoomin, J.Y. Huang, M. Bowman, M. Iovene, W. Sanseverino, P. Cavagnaro, M. Yildiz, A. Macko-Podgorni, E. Moranska, E. Grzebelus, D. Grzebelus, H. Ashrafi, Z.J. Zheng, S.F. Cheng, D. Spooner, A. Van Deynze, and P. Simon. 2016. A high-quality carrot genome assembly provides new insights into carotenoid accumulation and asterid genome evolution. Nature Genetics 48:657-+, <Go to ISI>://WOS:000376744200014.

Jaillon, O., J.-M. Aury, B. Noel, A. Policriti, C. Clepet, A. Casagrande, N. Choisne, S. Aubourg, N. Vitulo, and C. Jubin. 2007. The grapevine genome sequence suggests ancestral hexaploidization in major angiosperm phyla. nature 449:463.

Jarvis, D.E., Y.S. Ho, D.J. Lightfoot, S.M. Schmöckel, B. Li, T.J.A. Borm, H. Ohyanagi, K. Mineta, C.T. Michell, N. Saber, N.M. Kharbatia, R.R. Rupper, A.R. Sharp, N. Dally, B.A. Boughton, Y.H. Woo, G. Gao, E.G.W.M. Schijlen, X. Guo, A.A. Momin, S. Negrão, S. Al-Babili, C. Gehring, U. Roessner, C. Jung, K. Murphy, S.T. Arold, T. Gojobori, C.G.V.D. Linden, E.N. Van Loo, E.N. Jellen, P.J. Maughan, and M. Tester. 2017. The genome of Chenopodium quinoa. Nature 542:307-312, <https://dx.doi.org/10.1038/nature21370>.

Kawahara, Y., M. de la Bastide, J.P. Hamilton, H. Kanamori, W.R. McCombie, S. Ouyang, D.C. Schwartz, T. Tanaka, J. Wu, and S. Zhou. 2013. Improvement of the Oryza sativa Nipponbare reference genome using next generation sequence and optical map data. Rice 6:4.

Khattak, J.Z.K., A.M. Torp, and S.B. Andersen. 2006. A genetic linkage map of Spinacia oleracea and localization of a sex determination locus. Euphytica 148:311-318, <https://doi.org/10.1007/s10681-005-9031-1>.

Krzywinski, M., J. Schein, I. Birol, J. Connors, R. Gascoyne, D. Horsman, S.J. Jones, and M.A. Marra. 2009. Circos: an information aesthetic for comparative genomics. Genome research 19:1639-1645.

Kumar, S., G. Stecher, M. Suleski, and S.B. Hedges. 2017. TimeTree: a resource for timelines, timetrees, and divergence times. Molecular biology and evolution 34:1812-1819.

Li, H. 2016. Minimap and miniasm: fast mapping and de novo assembly for noisy long sequences. Bioinformatics 32:2103-2110.

Li, H., and R. Durbin. 2010. Fast and accurate long-read alignment with Burrows–Wheeler transform. Bioinformatics 26:589-595.

Li, L., S. Wang, H. Wang, S.K. Sahu, B. Marin, H. Li, Y. Xu, H. Liang, Z. Li, S. Cheng, T. Reder, Z. Çebi, S. Wittek, M. Petersen, B. Melkonian, H. Du, H. Yang, J. Wang, G.K.-S. Wong, X. Xu, X. Liu, Y. Van de Peer, M. Melkonian, and H. Liu. 2020. The genome of Prasinoderma coloniale unveils the existence of a third phylum within green plants. Nature Ecology & Evolution 4:1220-1231, <https://doi.org/10.1038/s41559-020-1221-7>.

Li, R., Y. Li, K. Kristiansen, and J. Wang. 2008. SOAP: short oligonucleotide alignment program. Bioinformatics 24:713-714, <http://bioinformatics.oxfordjournals.org/content/24/5/713.abstract>.

Myburg, A.A., D. Grattapaglia, G.A. Tuskan, U. Hellsten, R.D. Hayes, J. Grimwood, J. Jenkins, E. Lindquist, H. Tice, and D. Bauer. 2014. The genome of Eucalyptus grandis. Nature 510:356-362.

Osuna-Cruz, C.M., A. Paytuvi-Gallart, A. Di Donato, V. Sundesha, G. Andolfo, R. Aiese Cigliano, W. Sanseverino, and M.R. Ercolano. 2018. PRGdb 3.0: a comprehensive platform for prediction and analysis of plant disease resistance genes. Nucleic acids research 46:D1197-D1201.

R Development Core Team. R: a language and environment for statistical computing. R Foundation for Statistical Computing. 2015, Vienna, Austria.

Simillion, C., K. Vandepoele, M.C. Van Montagu, M. Zabeau, and Y. Van de Peer. 2002. The hidden duplication past of Arabidopsis thaliana. Proceedings of the National Academy of Sciences 99:13627-13632.

Stanke, M., O. Keller, I. Gunduz, A. Hayes, S. Waack, and B. Morgenstern. 2006. AUGUSTUS: ab initio prediction of alternative transcripts. Nucleic acids research 34:W435-W439.

Stoffel, K., H. van Leeuwen, A. Kozik, D. Caldwell, H. Ashrafi, X. Cui, X. Tan, T. Hill, S. Reyes-Chin-Wo, M.J. Truco, R.W. Michelmore, and A. Van Deynze. 2012. Development and application of a 6.5 million feature affymetrix genechip (R) for massively parallel discovery of single position polymorphisms in lettuce (Lactuca spp.). BMC Genomics 13:185, <http://www.ncbi.nlm.nih.gov/pubmed/22583801>.

Tang, H., V. Krishnakumar, J. Li, and X. Zhang. 2015. jcvi: JCVI utility libraries. Zenodo. (doi: 10.5281/zenodo. 31631).

Vij, S., H. Kuhl, I.S. Kuznetsova, A. Komissarov, A.A. Yurchenko, P. Van Heusden, S. Singh, N.M. Thevasagayam, S.R.S. Prakki, K. Purushothaman, J.M. Saju, J. Jiang, S.K. Mbandi, M. Jonas, A. Hin Yan Tong, S. Mwangi, D. Lau, S.Y. Ngoh, W.C. Liew, X. Shen, L.S. Hon, J.P. Drake, M. Boitano, R. Hall, C.-S. Chin, R. Lachumanan, J. Korlach, V. Trifonov, M. Kabilov, A. Tupikin, D. Green, S. Moxon, T. Garvin, F.J. Sedlazeck, G.W. Vurture, G. Gopalapillai, V. Kumar Katneni, T.H. Noble, V. Scaria, S. Sivasubbu, D.R. Jerry, S.J. O'Brien, M.C. Schatz, T. Dalmay, S.W. Turner, S. Lok, A. Christoffels, and L. Orbán. 2016. Chromosomal-Level Assembly of the Asian Seabass Genome Using Long Sequence Reads and Multi-layered Scaffolding. PLOS Genetics 12:e1005954, <https://dx.doi.org/10.1371/journal.pgen.1005954>.

Walker, B.J., T. Abeel, T. Shea, M. Priest, A. Abouelliel, S. Sakthikumar, C.A. Cuomo, Q. Zeng, J. Wortman, and S.K. Young. 2014. Pilon: an integrated tool for comprehensive microbial variant detection and genome assembly improvement. PloS one 9:e112963.

Wang, Y., H. Tang, J.D. DeBarry, X. Tan, J. Li, X. Wang, T.-h. Lee, H. Jin, B. Marler, and H. Guo. 2012. MCScanX: a toolkit for detection and evolutionary analysis of gene synteny and collinearity. Nucleic acids research 40:e49-e49.

Workman, R.E., A.M. Myrka, G.W. Wong, E. Tseng, K.C. Welch, Jr., and W. Timp. 2018. Single-molecule, full-length transcript sequencing provides insight into the extreme metabolism of the ruby-throated hummingbird Archilochus colubris. GigaScience 7, <https://doi.org/10.1093/gigascience/giy009>.

Wu, T.D., J. Reeder, M. Lawrence, G. Becker, and M.J. Brauer. 2016. GMAP and GSNAP for genomic sequence alignment: enhancements to speed, accuracy, and functionality, p. 283-334 Statistical genomics. Springer.

Xu, C., C. Jiao, H. Sun, X. Cai, X. Wang, C. Ge, Y. Zheng, W. Liu, X. Sun, Y. Xu, J. Deng, Z. Zhang, S. Huang, S. Dai, B. Mou, Q. Wang, Z. Fei, and Q. Wang. 2017. Draft genome of spinach and transcriptome diversity of 120 Spinacia accessions. Nat Commun 8:15275, <https://www.ncbi.nlm.nih.gov/pubmed/28537264>.

Zdobnov, E.M., and R. Apweiler. 2001. InterProScan–an integration platform for the signature-recognition methods in InterPro. Bioinformatics 17:847-848.
